# Development of a comprehensive set of tools for genome engineering in a cold- and thermo-tolerant *Kluyveromyces marxianus* yeast strain

**DOI:** 10.1038/s41598-017-08356-5

**Published:** 2017-08-21

**Authors:** Yumiko Nambu-Nishida, Keiji Nishida, Tomohisa Hasunuma, Akihiko Kondo

**Affiliations:** 1 Technology Research Association of Highly Efficient Gene Design (TRAHED), 7-1-49 Minatojimaminamimachi, Chuo-ku, Kobe, Hyogo, 650-0047 Japan; 2 Department of Chemical Science and Engineering, Graduate School of Engineering, Kobe University, 1-1 Rokkodai-cho, Nada-ku, Kobe, Hyogo 657-8501 Japan; 3 Graduate School of Science, Technology and Innovation, Kobe University, 1-1 Rokkodai-cho, Nada-ku, Kobe, Hyogo 657-8501 Japan; 4 Biomass Engineering Program, RIKEN, 1-7-22 Suehiro-cho, Tsurumi-ku, Yokohama, Kanagawa 230-0045 Japan

## Abstract

*Kluyveromyces marxianus*, a non-conventional thermotolerant yeast, is potentially useful for production of ethanol and other products. This species has a strong tendency to randomly integrate transforming DNA fragments, making necessary the development of more precise methods for gene targeting. In this study, we first demonstrated that *K. marxianus* NBRC1777 is cold-tolerant, and then established a highly efficient and precise technique for gene editing by introducing genes encoding deaminase-mediated targeted point mutagenesis (Target-AID) and clustered regularly interspaced short palindromic repeats (CRISPR) associated proteins (CRISPR-Cas9). We used Target-AID to introduce targeted point mutations that disrupted *Nej1* or *Dnl4*, genes that are involved in non-homologous end-joining (NHEJ). Both of the resulting mutant strains showed enhanced proportions of homology-mediated integration compared to the wild-type parent. In combination with target cleavage by CRISPR-Cas9, markerless integration was performed using short (~50 bp) flanking homologous sequences. Together, these tools render this species fully tractable for gene manipulation, permitting targeted genetic changes in the cold- and thermo-tolerant yeast *K. marxianus*.

## Introduction

The non-conventional yeast *Kluyveromyces marxianus* is known to be thermotolerant and Crabtree-negative. The latter term indicates that this species produces ethanol only under anaerobic conditions^1^; in contrast, Crabtree-positive yeasts such as *Saccharomyces cerevisiae* obligately produce ethanol both aerobically and anaerobically. Thus, Crabtree-negative yeasts are more suitable for the generation of various products other than ethanol because these strains do not undergo aerobic alcoholic fermentation, which consumes carbon resources^2^. The thermotolerance of *K. marxianus* also allows faster growth and production at higher temperatures, and some volatile products can be collected during synthesis more efficiently at higher temperature. Another benefit of the faster growth of *K. marxianus* at 37 °C is the ability to obtain single colonies after overnight incubation, while generally used *S. cerevisiae* strains take 2 to 3 days at 30 °C. Recently, the complete genome sequence for *K. marxianus* has been reported^3–5^.

Unlike *S. cerevisiae*, which demonstrates homologous recombination (HR) of transformed DNA, *K. marxianus* DMKU3-1042 tends to integrate DNA fragments randomly into its genome^1^. Such random integration is attributed to the high activity of the non-homologous end-joining (NHEJ) pathway^6^. *K. marxianus* NBRC1777 reportedly needed relatively large homology arms (641 and 1475 bp) of flanking DNA for insertion by HR^7^, while *S. cerevisiae* requires only ~50-bp homology arms, sequence lengths that can be added at the 5′ ends of PCR primers^8^.

Disruption of the NHEJ pathway in yeasts has been shown to suppress random integration events and therefore increase the success rate of HR in these organisms^6, 9^. The core NHEJ proteins are Ku70/Ku80, Lig4/Lif1, and Nej1^10–13^. In the first step, a stable heterodimer of Ku70/Ku80 binds to broken DNA ends and acts as a bridging complex. Lig4 and Lif1 also form a highly stable complex. Lig4 is an ATP-dependent double-strand break (DSB) repair DNA ligase and Lif1 is Lig4’s stabilizing, stimulating, and targeting co-factor^10^. In *S. cerevisiae*, deletion of *NEJ1* reduces NHEJ by 100-fold^9^.

Clustered regularly interspaced short palindromic repeats (CRISPR) associated proteins (CRISPR-Cas9) genome-editing tools have been applied in several yeast species, facilitating NHEJ-mediated insertion/deletion formation. HR-mediated integration following CRISPR-Cas9 editing has also been demonstrated, with efficiency varying depending on the yeast species^2, 14^. More recently, deaminase-mediated targeted point mutagenesis (Target-AID) has been performed in *S. cerevisiae*; which is a hybrid system of nuclease-deficient CRISPR-Cas9 and activation-induced cytidine deaminase (AID)^15^. When AID ortholog PmCDA1 was fused to C-terminus of nickase Cas9(D10A), C to T/G point mutation was induced within 3–5 bases window at the 5′ end of target sequence. Target-AID also appeared to be much less toxic compared to CRISPR-Cas9.

Here, we describe the development of a comprehensive set of genome engineering tools for use with *K. marxianus* NBRC1777, a strain that we show to be both cold- and thermo-tolerant.

## Results and Discussions

### Cold-tolerant yeast *K. marxianus* strain

**Figure 1:**
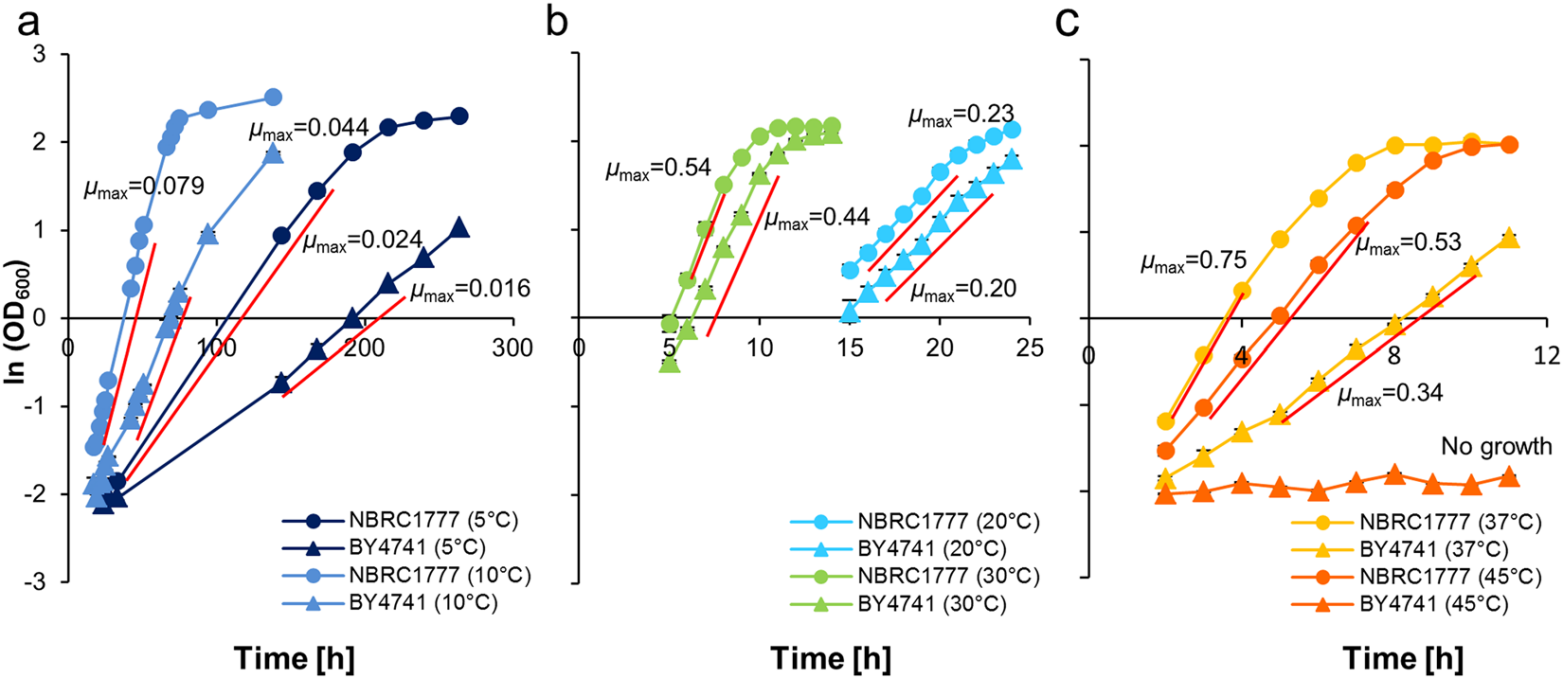
Cell growth of *K. marxianus* and *S. cerevisiae* at various temperatures. Cell growth was compared between *K. marxianus* NBRC1777 and *S. cerevisiae* BY4741 at 5, 10 °C (**a**), at 20, 30 °C (**b**), and at 37, 45 °C (**c**). The maximum specific growth rate (*µ*_max_) was determined as the slope of the mid-log phase of natural logarithm (ln) (OD_600_). Timeframes for each *µ*_max_ were as follows: at 5 °C from 33 h to 168 h and 144 h to 216 h, at 10 °C from 19 h to 48 h and 42 h to 75 h, at 20 °C from 16 h to 20 h and 16 h to 22 h, at 30 °C from 6 h to 8 h and from 6 h to 10 h, at 37 °C from 2 h to 4 h and 5 h to 10 h, 45 °C from 3 h to 7 h and no growth for *K. marxianus* and *S. cerevisiae*, respectively.

To characterize growth rates at various temperature, *K. marxianus* NBRC1777 and *S. cerevisiae* BY4741 were cultured at 5, 10, 20, 30, 37, and 45 °C (Fig. 1). The maximum specific growth rate (*µ*_max_) was higher for *K. marxianus* than for *S. cerevisiae* at all temperatures (Fig. 1), indicating that the *K. marxianus* is a fast-growing strain*. K. marxianus* showed best growth at 37 °C and was able to grow at 45 °C. The difference in the *µ*_max_ was also bigger at 10 °C (0.079/0.044) compared to the difference at 20 °C (0.23/0.20). Thus, in addition to the known thermotolerance of *K. marxianus* ^7^, strain NBRC1777 exhibited fast and robust growth at a wide range of temperatures, a feature that will be appealing for a variety of industrial applications.

### Isolation of Km*SNR52* promoter for expression of sgRNA for CRISPR-Cas9

To introduce the CRISPR system into *K. marxianus*, an efficient promoter for sgRNA expression was needed. As 5′-capped transcripts are not suitable for use as sgRNAs, PolII-driven promoters are not favoured. The *SNR52* promoter has been used to express functional sgRNAs in *S. cerevisiae* ^16^, so we obtained the *K. marxianus SNR52* promoter (KmP_*SNR52*_) as follows. The *K. marxianus SNR52* transcript sequence was first identified using the BLASTN search program with the *S. cerevisiae SNR52* transcript as a query. The region upstream of the putative *KmSNR52* transcript was then aligned with the Sc*SNR52* promoter; a 531-bp fragment was thereby defined as the putative KmP_*SNR52*_ (Supplementary Fig. S1).

We next constructed a vector for use as a shuttle vector by combining a *K. marxianus* autonomously replicating sequence (KmARS7)^17^ with a centromere sequence (KmCEN-D)^18^ and demonstrated (data not shown) that this plasmid is retained as an episome in *K. marxianus* NBRC1777. A human-optimized *Streptococcus pyogenes* (Sp) *cas9* ^16^ under the control of the constitutive *S. cerevisiae PDC1* promoter (ScP_*PDC1*_) was inserted into the shuttle vector, yielding a basal plasmid (Cas9_Base; Supplementary Fig. S2 and S3) capable of directing the expression of Cas9 in *K. marxianus*.

### Inactivation of the NHEJ pathway by Target-AID point mutation of NHEJ proteins

**Figure 2:**
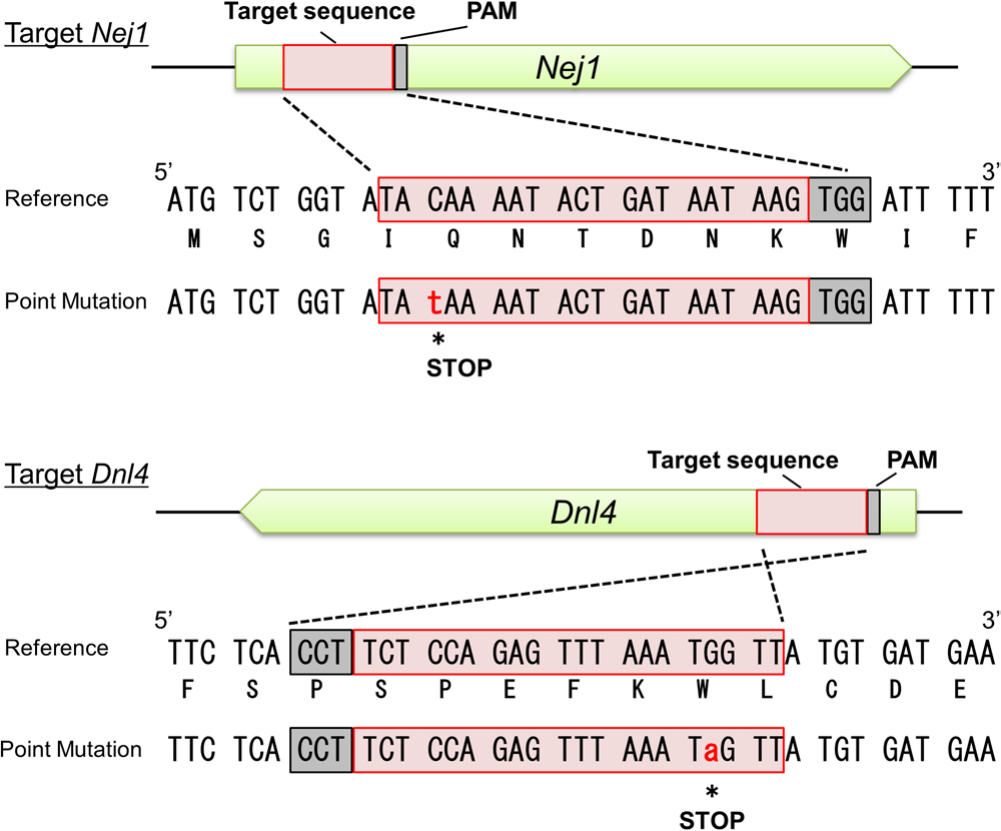
Induction of mutations by Target-AID. The *Nej1* and *Dnl4* target sites were selected for the introduction of stop codons by Target-AID. Red boxes indicate the target sites and grey boxes indicate the PAM sequences. Lower-case nucleotide in red font indicates the mutated base. The sequences of the induced mutations are shown in alignment with the reference sequences, with each predicted amino acid sequence below the respective nucleic acid sequence.

In *S. cerevisiae*, the *NEJ1* and *DNL4* genes encode core components of the proteins mediating NHEJ. By analogy to *S. cerevisiae*, disruption of the *K. marxianus Nej1* and *Dnl4* homologs are expected to yield more genetically tractable *K. marxianus* hosts with higher proportions of HR. Mutation of the *K. marxianus Nej1* and *Dnl4* genes identified by sequence similarity to the respective budding yeast genes was performed using targeted point mutagenesis by Target-AID in *K. marxianus*. A basal plasmid (nCas9-CDA_Base) was generated by constructing a variant of the Cas9_Base vector that encoded a nickase mutant (D10A) SpCas9 protein fused to the *Petromyzon marinus* (Pm)CDA1 cytidine deaminase^15^ (Supplementary Fig. S2 and S4). Following a previous study in *S. cerevisiae* ^9^, target sites in the *Nej1* and *Dnl4* genes of *K. marxianus* NBRC1777 were selected so as to introduce stop codons by causing C-to-T mutagenesis at 16 to 19 bp upstream of the sequences encoding protospacer adjacent motif (PAM) sequences for the target sequences. *K. marxianus* NBRC1777 cells were transformed with plasmids containing each targeting sequence (nCas9-CDA_target *Nej1* and nCas9-CDA_target *Dnl4*). G418-resistant colonies were selected; the *Nej1* and *Dnl4* genes of the transformants then were PCR amplified and subjected to sequence analysis to confirm the presence of the targeted lesions (Fig. 2). Both plasmids introduced stop codons as expected, providing null alleles of *Nej1* (*Nej1*°) and *Dnl4* (*Dnl4*°) with 1 of 8 (or 1 of 4) G418-resistant colonies contained the mutant allele, respectively. Thus, Target-AID was efficient enough to introduce targeted point mutation in *K. marxianus*.

### Conventional HR in NHEJ null mutants

**Figure 3:**
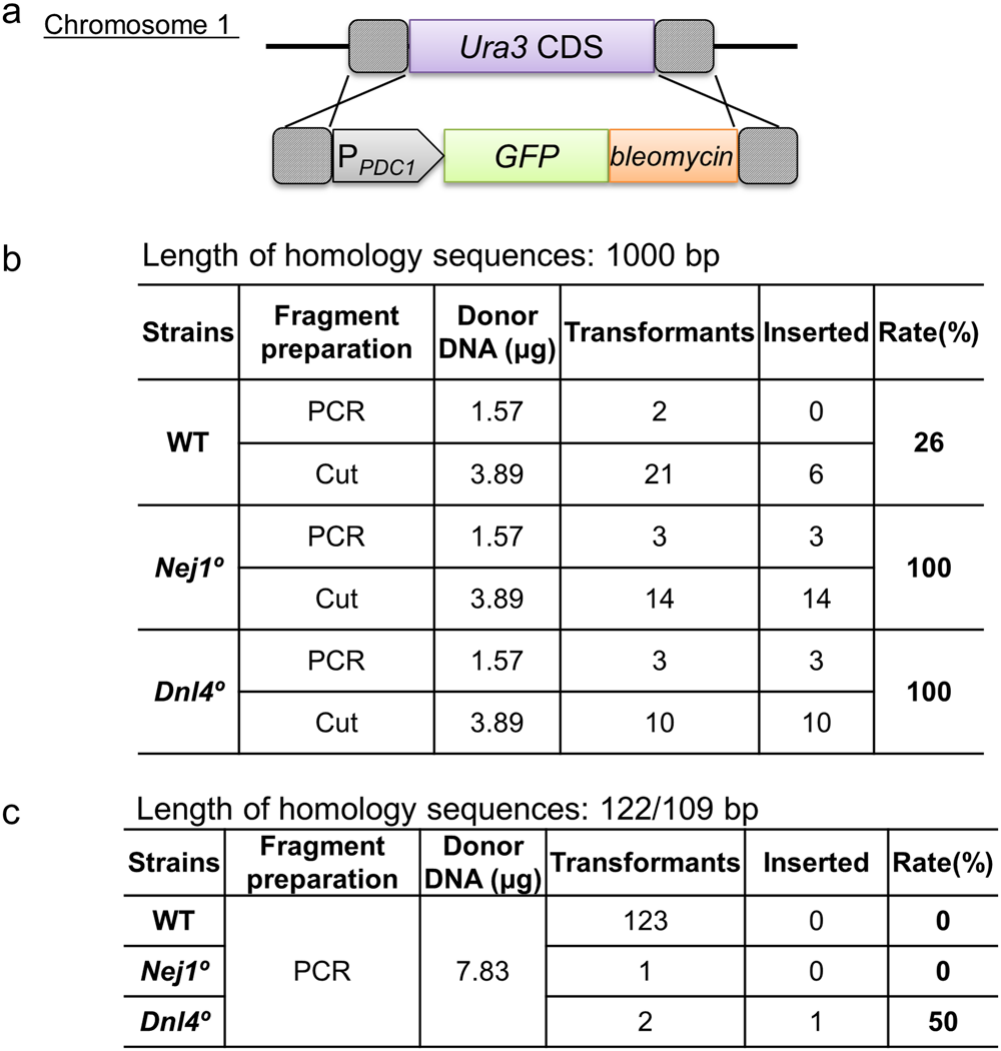
Effect of *Nej1°* and *Dnl4°* on homologous recombination. (**a**) Schematic of the transforming donor DNA fragment targeting the *Ura3* locus. Dashed boxes indicate homologous regions. (**b**), (**c**) Homologous recombination rates (5-FOA resistant (inserted)/zeocin resistant (transformants)). Fragments were prepared either by PCR or by restriction digestion of plasmid (Cut).

To investigate if these disruptions in genes with putative NHEJ activities increased the rate of HR in *K. marxianus*, cells of the *Nej1*° or *Dnl4*° mutants were transformed with DNA donor fragments with homology arms. Specifically, we employed a *GFP bleo* cassette (i.e., encoding green fluorescent protein (GFP) fused to a protein providing a selectable marker for bleomycin/zeocin resistance) flanked by *K. marxianus Ura3* sequences. Two separate constructs were used, one providing extended homology (1000-bp arms at both ends) and the second providing shorter homology (arms of 122 and 109 bp at the two ends). The fragments were expected to replace *Ura3* with the *GFP-bleo* cassette if HR was facilitated by mutation of genes of the NHEJ pathway. The cells were transformed and selected for zeocin-resistance; the resulting transformants were then screened for 5-fluoroorotic acid (5-FOA) resistance, which would be conferred by *Ura3* disruption. When using the construct providing homology arms of 1000 bp, 100% of the Zeo^R^ transformants of the *Nej1*° or *Dnl4*° strains (17/17 and 13/13, respectively) were 5-FOA^R^; in comparison, only 26% (6/23) of the Zeo^R^ transformants of the wild-type (WT) strain were 5-FOA^R^ (Fig. 3). When using equimolar quantities of the construct providing shorter homology arms (122 bp/109 bp), 123 Zeo^R^ transformants were obtained from the WT strain but no 5-FOA^R^ transformants were obtained. The *Nej1*° or *Dnl4*° strains yielded one and two Zeo^R^ transformants (respectively), of which no and one (respectively) were 5-FOA^R^ (i.e., disrupted at the targeted *Ura3* locus) (Fig. 3).

Apparently, less number of transformants were obtained from the mutants of genes involved in NHEJ pathway, presumably because random integration was severely suppressed, rather than by increasing the HR frequency. This decrease in NHEJ facilitates reliable HR at the targeted (by homology) locus, although the obtained efficiency may not be sufficient to perform HR with short homology arms (i.e., of the length that can be added by PCR primers).

### HR with CRISPR-Cas9

**Figure 4:**
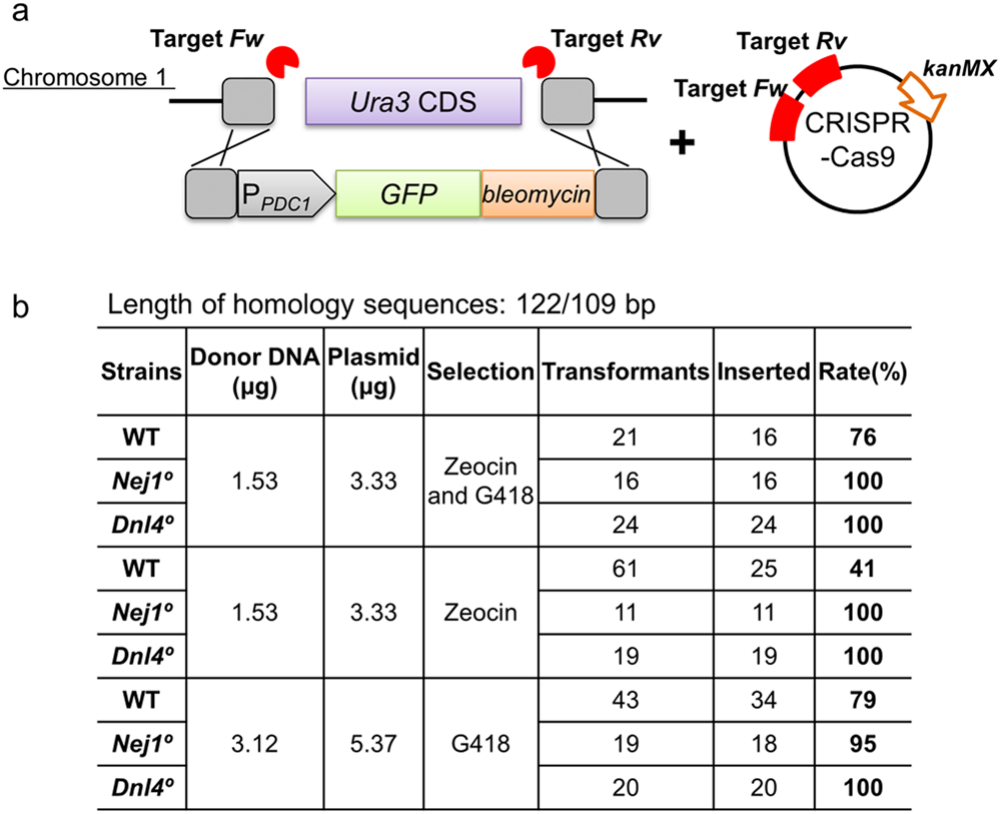
Homologous recombination facilitated by CRISPR-Cas9. (**a**) Schematic of the transforming donor DNA fragment targeting the *Ura3* locus and CRISPR-Cas9 plasmid targeting two sites (indicated by lightning bolts) flanking the *Ura3* coding sequences (CDS). Dashed boxes indicate homologous regions. Bottom panel indicates (**b**) Homologous recombination rate (5-FOA resistant (inserted)/zeocin and/or G418 resistant (transformants) * 100).

To boost the HR frequency, co-transformation with a construct encoding CRISPR-Cas9 was performed; the CRISPR system was expected to facilitate HR by providing cleavage of the targeted locus. A pair of target sites was selected at just inside of the homologous regions of *Ura3*. WT, *Nej1*°, and *Dnl4*° cells were transformed with the fragment with homology arms of 122 bp/109 bp (in combination with the Cas9 construct) and transformants were selected using either zeocin, (*Ura3*-targeting fragment), G418 (CRISPR-Cas9 vector), or both. For all three strains, a larger number of antibiotic-resistant transformants were obtained when the transformation mixes included the CRISPR-Cas9 construct. Among the antibiotic-resistant transformants, 41–79% of those derived from WT cells were 5-FOA^R^ (i.e., disrupted at *Ura3*), while 95–100% of those derived from the *Nej1*° and *Dnl4*° strains were 5-FOA^R^ (Fig. 4). These results indicated that target cleavage by Cas9 enhanced the frequency of HR sufficiently that even without selection by zeocin (i.e., without direct selection for the integration at *Ura3*), disruption of Ura3 was seen in virtually all transformants.

### Markerless integration with 50-bp homology arms and CRISPR-Cas9

**Figure 5:**
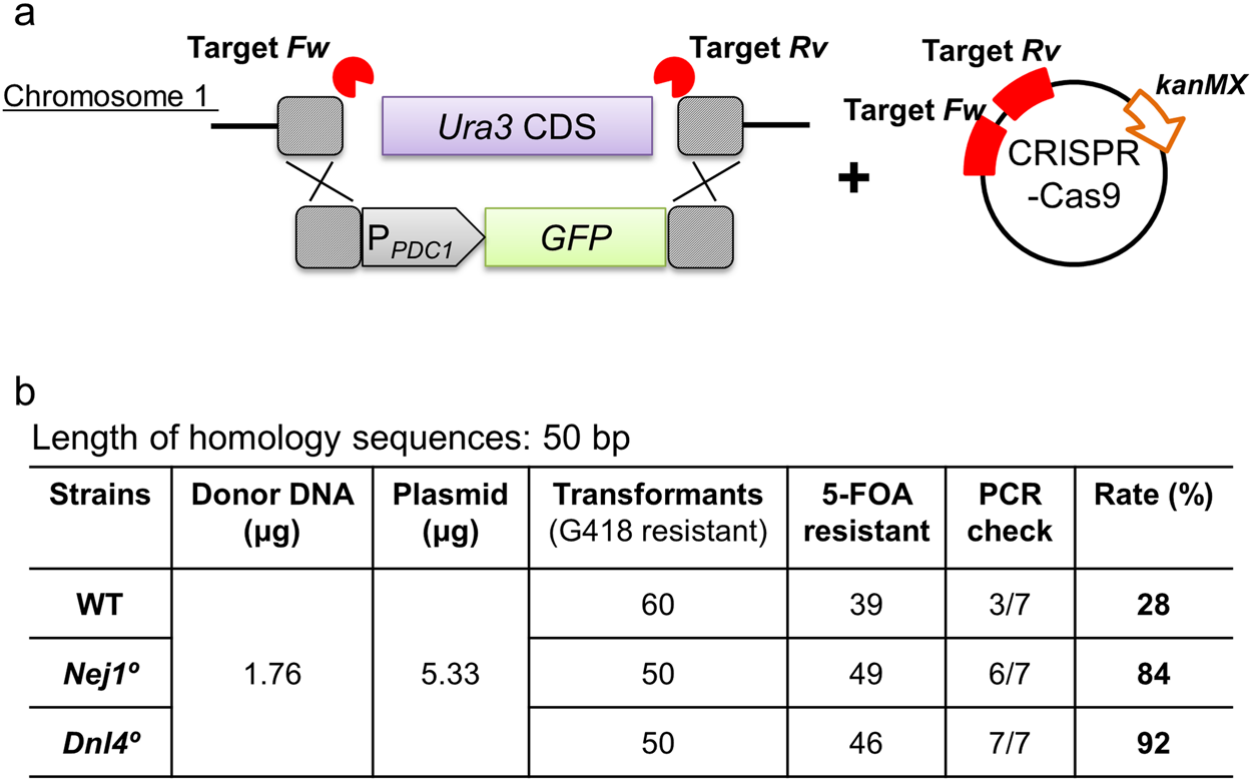
Markerless integration with short homologous arms. (**a**) Schematic of the transforming donor DNA fragment targeting the *Ura3* locus and the CRISPR-Cas9 plasmid targeting two sites (indicated by lightning bolts) flanking the *Ura3* CDS. Dashed boxes indicate homologous regions. (**b**) Homologous recombination rate ((5-FOA resistant * PCR check)/ transformants (G418 resistant) * 100).

**Figure 6:**
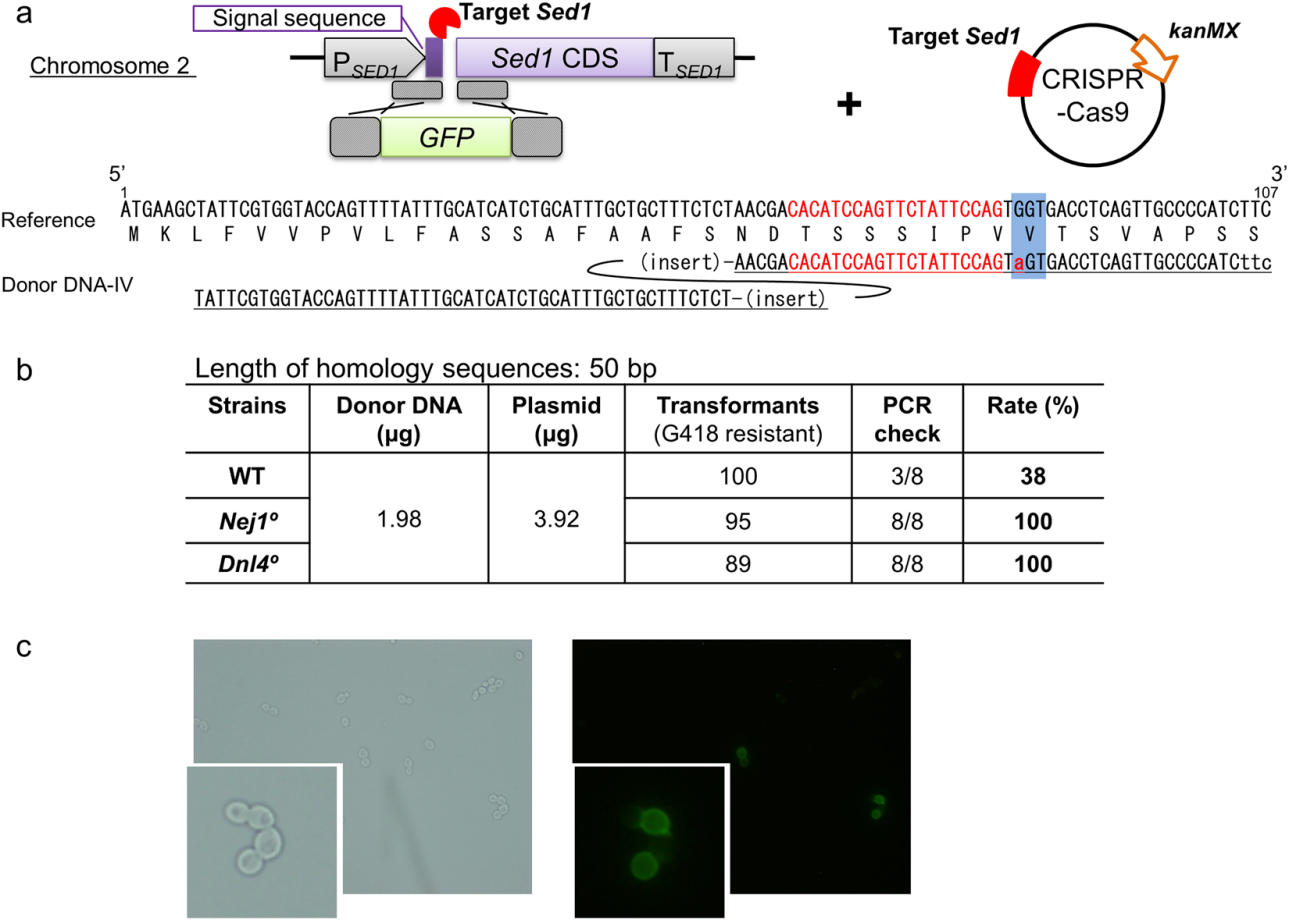
Direct fusion of GFP-coding sequence into a chromosomal locus. (**a**) Schematic showing integration of the GFP-coding fragment into the *Sed1* CDS. The CRISPR-Cas9 plasmid targeted (indicated by lightning bolts) the end of the signal peptide-coding region of *Sed1*. Dashed boxes indicate homologous regions. Left arm is homologous to the end of to the signal peptide-coding sequence (bp 8 to 57 with respect to the start of the *Sed1* coding sequence). Right arm is homologous to *Sed1* sequence (bp 58 to 107 with respect to the start of the *Sed1* coding sequence) with substitution at bp 84 (G to A; indicated by lower-case letter in red font) to eliminate a PAM. (**b**) The successful integration rate was calculated as PCR check * 100. (**c**) Representative microscopic image of positive transformants (*Nej1°*/GFP_Sed1) under bright-field (left) and fluorescent (right) illumination. Insets show 3-fold magnified images.

Markerless integration was attempted by co-transformation using the combination of a donor DNA template containing a *GFP* expression cassette flanked by 50-bp homology arms along with a CRISPR-Cas9-encoding construct. Transformants were selected using G418 and then screened by both the 5-FOA test and PCR analysis. We observed successful integration (i.e., disruption of the *Ura3* target locus in G418^R^ transformants) at rates of 28%, 84%, and 92% in the WT, *Nej1*°, and *Dnl4*° backgrounds, respectively (Fig. 5). Taking advantage of the markerless integration, the GFP-encoding sequence was directly inserted into the coding sequence of the endogenous *Sed1* gene so as to encode a GFP-Sed1 fusion protein preceded by the Sed1 localization peptide. A single cleavage site for CRISPR-Cas9 within this target gene was designed in such a way that the site would not be retained after successful integration. Short (50-bp) homology arms flanking the target site were added to either end of the GFP-encoding sequence by PCR. Transformants were selected by G418 and then screened by PCR, revealing gene replacement at the *Sed1* target locus at 38%, 100%, and 100% success rates in the WT, *Nej1*°, and *Dnl4*° backgrounds, respectively (Fig. 6). When viewed by fluorescence microscopy, cells harboring the targeted integration exhibited peripheral localization of GFP-Sed1, consistent with the localization previously reported for the equivalent construct in *S. cerevisiae* ^19^. Thus, this method can be applied for scar-less genome editing in *K. marxianus*.

### Assembly and integration of multiple fragments without markers

**Figure 7:**
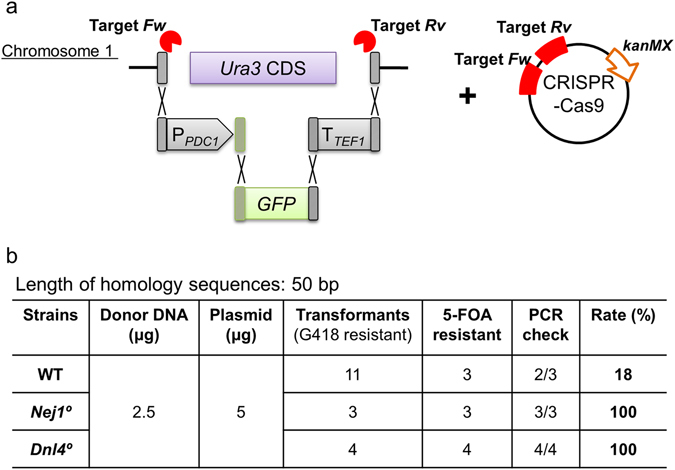
*In vivo* assembly and integration of three DNA fragments with 50 bp overlaps. (**a**) Schematic of the three transforming donor DNA fragments replacing the *Ura3* locus along with the CRISPR-Cas9 plasmid targeting two sites (indicated by lightning bolts) flanking the *Ura3* CDS. Dashed boxes indicate homologous regions. Bottom panel shows (**b**) Homologous recombination rate ((5-FOA resistant * PCR check)/ transformants (G418 resistant) * 100).

*In vivo* assembly and integration of multiple fragments was performed using three DNA fragments, each containing 50 bp overlap with the adjacent fragments or target sites, such that the entire construct was designed to replace *Ura3* (Fig. 7). Compared to single PCR product donor constructs, a smaller number of transformants was obtained by G418 selection when using the multiple-fragment DNA as the donor. Nonetheless, the WT, *Nej1*°, and *Dnl4*° strains showed integration efficiencies of 18% (2/11), 100% (3/3), and 100% (4/4), respectively. This assembly and integration method is expected to allow combinatorial integration of gene cassettes using (for example) combinations of various promoters and coding regions.

In summary, targeted point mutagenesis (by Target-AID) and markerless CRISPR-mediated integration were demonstrated in *K. marxianus* using a fast-growing strain that was shown to exhibit both thermo- and cold-tolerance. Mutation of *Nej1* or *Dnl4* loci encoding homologs of NHEJ proteins, enhanced the proportion of HR events. These genome-engineering tools are expected to greatly facilitate industrial use of *K*. marxianus, a prototype for the class of underexploited non-conventional yeasts.

## Methods

### Strains and culture conditions

*S. cerevisiae* and *K. marxianus* strains used in this study are listed in Table 1. Yeast cells were grown in YPD medium (10 g/L yeast extract, 20 g/L peptone, and 20 g/L glucose) at 30 °C unless otherwise noted. *Escherichia coli* strain DH5α (Toyobo, Osaka, Japan) which was used for cloning, was grown in Luria-Bertani (LB) medium (10 g/L peptone, 5 g/L yeast extract, and 5 g/L sodium chloride) supplemented with 100 mg/L ampicillin at 37 °C.

**Table 1:** Plasmids and strains used in this study.

Plasmids and strains	Genotype	Reference
Plasmids		
nCas9-CDA_Base	ScP_*PDC1*__nCas9_T_*TDH3*_, KmARS7, Km CEN D, *kanMX*, ori, and AmpR	This study
nCas9-CDA_target *Nej1*	KmP_*SNR52*__target_Nej1_sgRNA_T_*sup4*_ cassette in nCas9-CDA_Base	This study
nCas9-CDA_target *Dnl4*	KmP_*SNR52*__target_Dnl4_sgRNA_T_*sup4*_ cassette in nCas9-CDA_Base	This study
Cas9_Base	ScP_*PDC1*__Cas9_T_*TDH3*_, KmARS7, Km CEN D, *kanMX*, ori, and AmpR	This study
Cas9_Base_target_*Ura3*_Fw	KmP_*SNR52*__target_Ura3_Fw_sgRNA_T_*sup4*_ cassette in Cas9_Base	This study
Cas9_Base_target _Ura3_Fw + Rv	KmP_*SNR52*__target_Ura3_Fw_sgRNA_T_*sup4*_ cassette and KmP_*SNR52*__target_Ura3_Rv_sgRNA_T_*sup4*_ cassette in Cas9_Base	This study
Cas9_Base_target *Sed1*	KmP_*SNR52*__target_Sed1_sgRNA_T_*sup4*_ cassette in Cas9_Base	This study
TOPO/F7	ScP_*PDC1*__EGFP_bleomycin on both sides Ura3 homologous sequence cassette in pCR^TM^4Blunt-TOPO^®^	This study
Strains		
*S. cerevisiae*		
BY4741	*MAT*a *his3*Δ*1 leu2*Δ0 *met15*Δ0 *ura3*Δ0	ATCC
*K. marxianus*		
NBRC1777	Wild-type	NITE Biological Resource Center, Japan
*Nej1*°	*Nej1*disrupted by C to T point mutation at position 13	This study
*Dnl4*°	*Dnl4* disrupted by G to A point mutation at position 44	This study

### Growth measurements

*S. cerevisiae* BY4741 and *K. marxianus* NBRC1777 strains were grown in YPD medium overnight at 30 °C by shaking in test tubes. The cells were inoculated into 5 mL of YPD medium at a starting optical density at 600 nm (OD_600_) of 0.05, and cultures were cultivated at 5, 10, 20, 30, 37, or 45 °C. Cell growth was tracked by measuring OD_600_ using a UV mini spectrophotometer (Shimadzu, Kyoto, Japan). The *µ*_max_ was determined as the slope of the log phase of natural logarithm (ln) transformation of the linear region.

### Construction of basal genome-editing plasmids

The vector sequences are provided in Supplementary Fig. S3 and S4. Vector assembly was performed by PCR using the In-fusion cloning method (Takara Bio, Shiga, Japan). The vector backbone was designed to contain KmARS7, KmCEN-D and selectable marker *kanMX*, which encodes G418 resistance. For CRISPR-Cas9 (Cas9_Base) and Target-AID (nCas9-CDA_Base) vectors, a gene encoding human-optimized nCas9-PmCDA1 or Cas9^15, 16^ (respectively) was placed between ScP_*PDC1*_ and the *TDH3* terminator (ScT_*TDH3*_) on the vector backbone.

### Construction of target sequence-containing plasmids

To generate the sgRNA expression casettes, the *K. marxianus SNR52* promoter (KmP_*SNR52*_) (531 bp; identified by alignment with the *S. cerevisiae* gene) was amplified by PCR using the combination of the forward primer (P_Km01-013) and each reverse primer (5′-CTAGCTCTAAAAC- reverse complement of target sequence-GATTCGAACTGCGGACGTTG-3′). The sgRNA scaffold and the *SUP4* terminator were amplified by PCR from plasmid pRS426-SNR52p-gRNA.CAN1.Y-SUP4t^16^ using the primers P_Km01-014 and P_Km01-015 (Supplementary Table S1). The two fragments were fused by overlap extension PCR using the primers P_Km01-013 and P_Km01-015 to yield each target-containing sgRNA cassette (Supplementary Fig. S1). Each cassette was digested with restriction enzymes NotI and NheI and ligated into NotI-, SpeI-digested nCas9-CDA_Base or Cas9_Base plasmid.

### Transformation

*K. marxianus* cells were transformed by the lithium acetate method^20^. After transformation, cells were plated on YPD containing appropriate selection reagents (100 μg/mL G418 and/or 50 μg/mL zeocin) and grown for one day. For the 5-FOA test, cells were streaked onto YPD containing 3 mg/mL 5-FOA, incubated overnight, and assessed for growth. Colony PCR was performed to check fragment size and to obtain DNA for Sanger sequencing using a 3130xL Genetic Analyzer (Applied Biosystems, CA, USA).

### Donor DNA templates

Each donor DNA template was constructed by PCR and cloned into the plasmid TOPO/F7 using the Zero Blunt^®^ TOPO^®^ PCR Cloning Kit (Invitrogen, CA, USA) (Fig. 5). Each cloned donor DNA was either excised by restriction enzymes or amplified by PCR using the respective primer pair (Supplementary Table S1), and the resulting fragment was purified by agarose gel electrophoresis before use in transformation.

## Electronic supplementary material

Supplementary Information
