# Supplementary material for: Development of a comprehensive set of tools for genome engineering in a cold- and thermo-tolerant Kluyveromyces marxianus yeast strain
Source: Sci Rep. 2017 Aug 21;7:8993. doi: 10.1038/s41598-017-08356-5 (PMC5566861; doi:10.1038/s41598-017-08356-5)
Supplement: Supplementary file 1 — Supplementary Information [file 41598_2017_8356_MOESM1_ESM.pdf]

Supplementary Information

Development of a comprehensive set of tools for genome engineering in a cold- and thermo-tolerant *Kluyveromyces marxianus* yeast strain

Yumiko Nambu-Nishida[1,2], Keiji Nishida[3], Tomohisa Hasunuma[3], Akihiko Kondo[2, 3,4,]*

[1]Technology Research Association of Highly Efficient Gene Design (TRAHED), 7-1-49 Minatojimaminamimachi, Chuo-ku, Kobe 650-0047, Japan

[2]Department of Chemical Science and Engineering, Graduate School of Engineering, Kobe University, 1-1 Rokkodai-cho, Nada-ku, Kobe, Hyogo 657-8501, Japan

[3]Graduate School of Science, Technology and Innovation, Kobe University, 1-1 Rokkodai-cho, Nada-ku, Kobe, Hyogo 657-8501, Japan

[4] Biomass Engineering Program, RIKEN, 1-7-22 Suehiro-cho, Tsurumi-ku, Yokohama, Kanagawa 230-0045, Japan

*akondo@kobe-u.ac.jp

AAGCTAGCTCTAGATGCCGCAAATAGGGCAGGCGATATACAAGGCGGTATACAGGGCGGTATACAAGGCG
TATGCCAGACGGTATGCCTGGAGGCATTGTCAGGAGGCATTGTCAGGAGGCATTGCCGGGCGAAATCGCC
AGCTTGAGTATGGCCCCAGAGGGAAGAAAAAACAGTTACCCAGGTGTGTGCCCCTGTATTACCCTGACTT
TGAAATGTACGCGAAGTACACTTCGTGCAGGGTGATGATGCTCGTATCAGGTGGACTTGGCGGACACGGT
ACGGTACGGTCCCCTTTTTTTATTTTTTCTTTTGGGAATGAGAAGGCCACGTGACGTTACCCTGGCCCTG
TGTTTCCCCCCTCGCATCTTGTACTGCCTTAGGACCCCTCGTAGTGTAGCGGTCCGGGCCCCCAGATTGC
GATGTGTTTCGAGAAAAACAACAAAAAAAAATAAGTTGAAAAGCGCGGATCAGGCGGTCCGCCCGGAGTGC
GCGCGCTGTATTCTCGCCTGACAGGGCGGGATTCGCAACGTCCGCAGTTCGAATCNNNNNNNNNNNNNNN
NNNNNGTTTTAGAGCTAGAAATAGCAAGTTAAAATAAGGCTAGTCCGTTATCAACTTGAAAAAGTGGCAC
CGAGTCGGTGGTGCTTTTTTTTGTTTTTTATGACTAGTATGCGGCCGCGAATTCAGG

Supplementary Figure S1 - Sequence of target-containing sgRNA cassette.

"N" indicates target specific sequence. This sequence indicates as follows: Purple: KmP$_{SNR52}$, Gray: sgRNA, Green: Sup4 terminator.

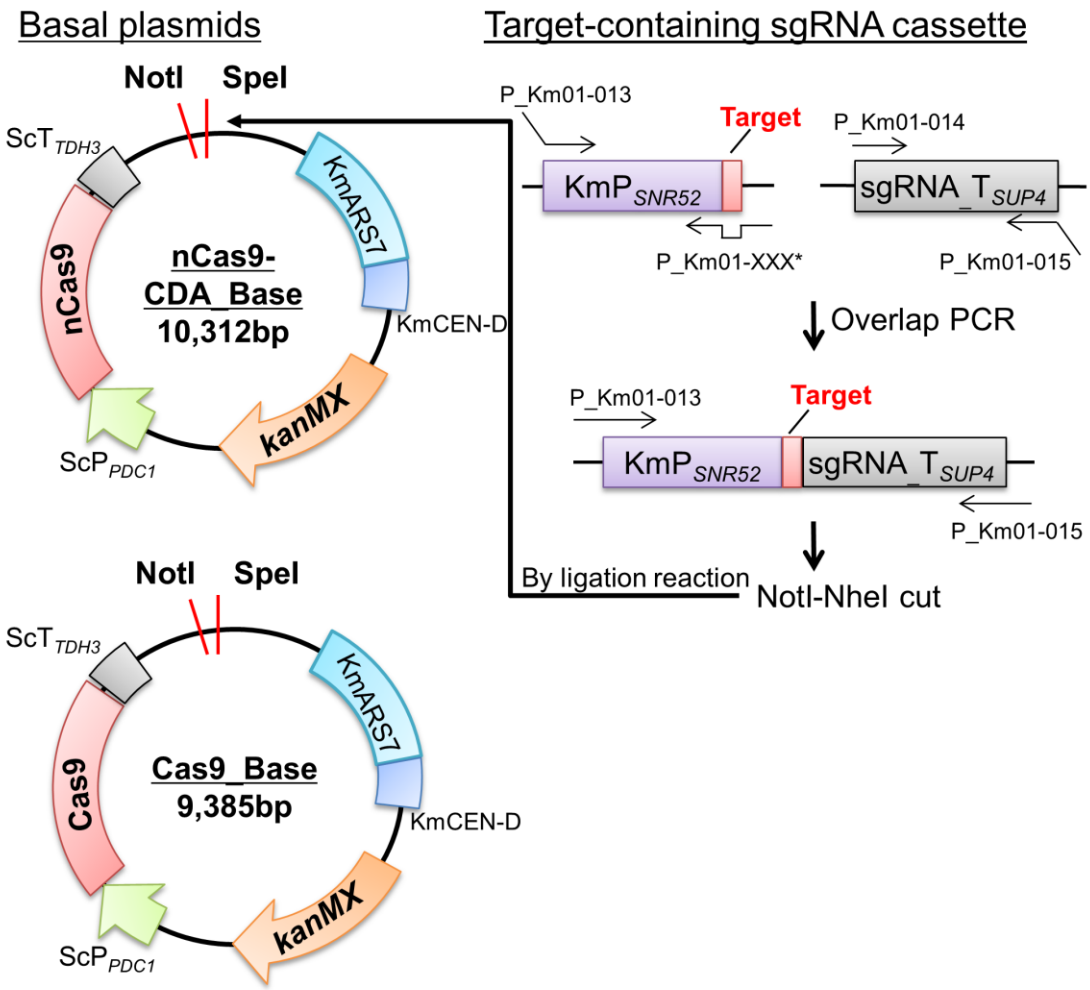

Supplementary Figure S2 - Construction of genome-editing plasmids.

Constructed basal plasmids nCas9-CDA_Base and Cas9_Base are shown on the left. Target-containing sgRNA cassettes were constructed by overlap PCR using target sequence-containing primers (P_Km01-XXX, where XXX was a 3-digit number as shown in the figure) and then inserted into the basal plasmids at NotI and SpeI/NheI restriction enzyme sites.

AAAAATGAAGTTTTAAATCAATCTAAAGTATATATGAGTAAACTTGGTCTGACAGTTACCAATGCTTAAT
CAGTGAGGCACCTATCTCAGCGATCTGTCTATTTCGTTCATCCATAGTTGCCTGACTCCCCGTCGTGTAG
ATAACTACGATACGGGAGGGCTTACCATCTGGCCCCAGTGCTGCAATGATACCGCGAGAACCACGCTCAC
CGGCTCCAGATTTATCAGCAATAAACCAGCCAGCCGGAAGGGCCGAGCGCAGAAGTGGTCCTGCAACTTT
ATCCGCCTCCATCCAGTCTATTAATTGTTGCCGGGAAGCTAGAGTAAGTAGTTCGCCAGTTAATAGTTTG
CGCAACGTTGTTGCCATTGCTACAGGCATCGTGGTGTCACGCTCGTCGTTTGGTATGGCTTCATTCAGCT
CCGGTTCCCAACGATCAAGGCGAGTTACATGATCCCCCATGTTGTGCAAAAAAGCGGTTAGCTCCTTCGG
TCCTCCGATCGTTGTCAGAAGTAAGTTGGCCGCAGTGTTATCACTCATGGTTATGGCAGCACTGCATAAT
TCTCTTACTGTCATGCCATCCGTAAGATGCTTTTCTGTGACTGGTGAGTACTCAACCAAGTCATTCTGAG
AATAGTGTATGCGGCGACCGAGTTGCTCTTGCCCGGCGTCAATACGGGATAATACCGCGCCACATAGCAG
AACTTTAAAAGTGCTCATCATTGGAAAACGTTCTTCGGGGCGAAAACTCTCAAGGATCTTACCGCTGTTG
AGATCCAGTTCGATGTAACCCACTCGTGCACCCAACTGATCTTCAGCATCTTTTACTTTCACCAGCGTTT
CTGGGTGAGCAAAAACAGGAAGGCAAAATGCCGCAAAAAAGGGAATAAGGGCGACACGGAAATGTTGAAT
ACTCATACTCTTCCTTTTTCAATATTATTGAAGCATTTATCAGGGTTATTGTCTCATGAGCGGATACATA
TTTGAATGTATTTAGAAAAATAAACAAATAGGGGTTCCGCGTTAATTAATTGAGATAAGCACACTGCACC
CATACCTTCCTTAAAAACGTAGCTTCCAGTTTTTGGTGGTTCCGGCTTCCTTCCCGATTCCGCCCGCTAA
ACGCATATTTTTGTTGCCTGGTGGCATTTGCAAAATGCATAACCTATGCATTTAAAAGATTATGTATGCT
CTTCTGACTTTTCGTGTGATGAGGCTCGTGGAAAAAATGAATAATTTATGAATTTGAGAACAATTTTGTG
TTGTTACGGTATTTTACTATGGAATAATCAATCAATTGAGGATTTTATGCAAATATCGTTTGAATATTTT
TCCGACCCTTTGAGTACTTTTCTTCATAATTGCATAATATTGTCCGCTGCCCCTTTTTCTGTTAGACGGT
GTCTTGATCTACTTGCTATCGTTCAACACCACCTTATTTTCTAACTATTTTTTTTTTAGCTCATTTGAAT
CAGCTTATGGTGATGGCACATTTTTGCATAAACCTAGCTGTCCTCGTTGAACATAGGAAAAAAAAATATA
TAAACAAGGCTCTTTCACTCTCCTTGCAATCAGATTTGGGTTTGTTCCCTTTATTTTCATATTTCTTGTC
ATATTCCTTTCTCAATTATTATTTTCTACTCATAACCTCACGCAAAATAACACAGTCAAATCAATCAAAG
CTAGCGAAATGGCACCGAAGAAGAAGCGTAAAGTCGGAATCCACGGAGTTCCTGCGGCAATGGACAAGAA
GTACTCCATTGGGCTCGATATCGGCACAAACAGCGTCGGTTGGGCCGTCATTACGGACGAGTACAAGGTG
CCGAGCAAAAAATTCAAAGTTCTGGGCAATACCGATCGCCACAGCATAAAGAAGAACCTCATTGGCGCCC
TCCTGTTCGACTCCGGGGAGACGGCCGAAGCCACGCGGCTCAAAAGAACAGCACGGCGCAGATATACCCG
CAGAAAGAATCGGATCTGCTACCTGCAGGAGATCTTTAGTAATGAGATGGCTAAGGTGGATGACTCTTTC
TTCCATAGGCTGGAGGAGTCCTTTTTGGTGGAGGAGGATAAAAAGCACGAGCGCCACCCAATCTTTGGCA
ATATCGTGGACGAGGTGGCGTACCATGAAAAGTACCCAACCATATATCATCTGAGGAAGAAGCTTGTAGA
CAGTACTGATAAGGCTGACTTGCGGTTGATCTATCTCGCGCTGGCGCATATGATCAAATTTCGGGGACAC

TTCCTCATCGAGGGGGACCTGAACCCAGACAACAGCGATGTCGACAAACTCTTTATCCAACTGGTTCAGA
CTTACAATCAGCTTTTCGAAGAGAACCCGATCAACGCATCCGGAGTTGACGCCAAAGCAATCCTGAGCGC
TAGGCTGTCCAAATCCCGGCGGCTCGAAAACCTCATCGCACAGCTCCCTGGGGAGAAGAAGAACGGCCTG
TTTGGTAATCTTATCGCCCTGTCACTCGGGCTGACCCCCAACTTTAAATCTAACTTCGACCTGGCCGAAG
ATGCCAAGCTTCAACTGAGCAAAGACACCTACGATGATGATCTCGACAATCTGCTGGCCCAGATCGGCGA
CCAGTACGCAGACCTTTTTTTGGCGGCAAAGAACCTGTCAGACGCCATTCTGCTGAGTGATATTCTGCGA
GTGAACACGGAGATCACCAAAGCTCCGCTGAGCGCTAGTATGATCAAGCGCTATGATGAGCACCACCAAG
ACTTGACTTTGCTGAAGGCCCTTGTCAGACAGCAACTGCCTGAGAAGTACAAGGAAATTTTCTTCGATCA
GTCTAAAAATGGCTACGCCGGATACATTGACGGCGGAGCAAGCCAGGAGGAATTTTACAAATTTATTAAG
CCCATCTTGGAAAAAATGGACGGCACCGAGGAGCTGCTGGTAAAGCTTAACAGAGAAGATCTGTTGCGCA
AACAGCGCACTTTCGACAATGGAAGCATCCCCCACCAGATTCACCTGGGCGAACTGCACGCTATCCTCAG
GCGGCAAGAGGATTTCTACCCCTTTTTGAAAGATAACAGGGAAAAGATTGAGAAAATCCTCACATTTCGG
ATACCCTACTATGTAGGCCCCCTCGCCCGGGGAAATTCCAGATTCGCGTGGATGACTCGCAAATCAGAAG
AGACCATCACTCCCTGGAACTTCGAGGAAGTCGTGGATAAGGGGGCCTCTGCCCAGTCCTTCATCGAAAG
GATGACTAACTTTGATAAAAATCTGCCTAACGAAAAGGTGCTTCCTAAACACTCTCTGCTGTACGAGTAC
TTCACAGTTTATAACGAGCTCACCAAGGTCAAATACGTCACAGAAGGGATGAGAAAGCCAGCATTCCTGT
CTGGAGAGCAGAAGAAAGCTATCGTGGACCTCCTCTTCAAGACGAACCGGAAAGTTACCGTGAAACAGCT
CAAAGAAGACTATTTCAAAAAGATTGAATGTTTCGACTCTGTTGAAATCAGCGGAGTGGAGGATCGCTTC
AACGCATCCCTGGGAACGTATCACGATCTCCTGAAAATCATTAAAGACAAGGACTTCCTGGACAATGAGG
AGAACGAGGACATTCTTGAGGACATTGTCCTCACCCTTACGTTGTTTGAAGATAGGGAGATGATTGAAGA
ACGCTTGAAAACTTACGCTCATCTCTTCGACGACAAAGTCATGAAACAGCTCAAGAGGCGCCGATATACA
GGATGGGGGCGGCTGTCAAGAAAACTGATCAATGGGATCCGAGACAAGCAGAGTGGAAAGACAATCCTGG
ATTTTCTTAAGTCCGATGGATTTGCCAACCGGAACTTCATGCAGTTGATCCATGATGACTCTCTCACCTT
TAAGGAGGACATCCAGAAAGCACAAGTTTCTGGCCAGGGGGACAGTCTTCACGAGCACATCGCTAATCTT
GCAGGTAGCCCAGCTATCAAAAAGGGAATACTGCAGACCGTTAAGGTCGTGGATGAACTCGTCAAAGTAA
TGGGAAGGCATAAGCCCGAGAATATCGTTATCGAGATGGCCCGAGAGAACCAAACTACCCAGAAGGGACA
GAAGAACAGTAGGGAAAGGATGAAGAGGATTGAAGAGGGTATAAAAGAACTGGGGTCCCAAATCCTTAAG
GAACACCCAGTTGAAAACACCCAGCTTCAGAATGAGAAGCTCTACCTGTACTACCTGCAGAACGGCAGGG
ACATGTACGTGGATCAGGAACTGGACATCAATCGGCTCTCCGACTACGACGTGGATCATATCGTGCCCCA
GTCTTTTCTCAAAGATGATTCTATTGATAATAAAGTGTTGACAAGATCCGATAAAAATAGAGGGAAGAGT
GATAACGTCCCCTCAGAAGAAGTTGTCAAGAAAATGAAAAATTATTGGCGGCAGCTGCTGAACGCCAAAC
TGATCACACAACGGAAGTTCGATAATCTGACTAAGGCTGAACGAGGTGGCCTGTCTGAGTTGGATAAAGC

CGGCTTCATCAAAAGGCAGCTTGTTGAGACACGCCAGATCACCAAGCACGTGGCCCAAATTCTCGATTCA
CGCATGAACACCAAGTACGATGAAAATGACAAACTGATTCGAGAGGTGAAAGTTATTACTCTGAAGTCTA
AGCTGGTCTCAGATTTCAGAAAGGACTTTCAGTTTTATAAGGTGAGAGAGATCAACAATTACCACCATGC
GCATGATGCCTACCTGAATGCAGTGGTAGGCACTGCACTTATCAAAAAATATCCCAAGCTTGAATCTGAA
TTTGTTTACGGAGACTATAAAGTGTACGATGTTAGGAAAATGATCGCAAAGTCTGAGCAGGAAATAGGCA
AGGCCACCGCTAAGTACTTCTTTTACAGCAATATTATGAATTTTTTCAAGACCGAGATTACACTGGCCAA
TGGAGAGATTCGGAAGCGACCACTTATCGAAACAAACGGAGAAACAGGAGAAATCGTGTGGGACAAGGGT
AGGGATTTCGCGACAGTCCGGAAGGTCCTGTCCATGCCGCAGGTGAACATCGTTAAAAAGACCGAAGTAC
AGACCGGAGGCTTCTCCAAGGAAAGTATCCTCCCGAAAAGGAACAGCGACAAGCTGATCGCACGCAAAAA
AGATTGGGACCCCAAGAAATACGGCGGATTCGATTCTCCTACAGTCGCTTACAGTGTACTGGTTGTGGCC
AAAGTGGAGAAAGGGAAGTCTAAAAAACTCAAAAGCGTCAAGGAACTGCTGGGCATCACAATCATGGAGC
GATCAAGCTTCGAAAAAAACCCCATCGACTTTCTCGAGGCGAAAGGATATAAAGAGGTCAAAAAAGACCT
CATCATTAAGCTTCCCAAGTACTCTCTCTTTGAGCTTGAAAACGGCCGGAAACGAATGCTCGCTAGTGCG
GGCGAGCTGCAGAAAGGTAACGAGCTGGCACTGCCCTCTAAATACGTTAATTTCTTGTATCTGGCCAGCC
ACTATGAAAAGCTCAAAGGGTCTCCCGAAGATAATGAGCAGAAGCAGCTGTTCGTGGAACAACACAAACA
CTACCTTGATGAGATCATCGAGCAAATAAGCGAATTCTCCAAAAGAGTGATCCTCGCCGACGCTAACCTC
GATAAGGTGCTTTCTGCTTACAATAAGCACAGGGATAAGCCCATCAGGGAGCAGGCAGAAAACATTATCC
ACTTGTTTACTCTGACCAACTTGGGCGCGCCTGCAGCCTTCAAGTACTTCGACACCACCATAGACAGAAA
GCGGTACACCTCTACAAAGGAGGTCCTGGACGCCACACTGATTCATCAGTCAATTACGGGGCTCTATGAA
ACAAGAATCGACCTCTCTCAGCTCGGTGGAGACAGCAGGGCTGACCCCAAGAAGAAGAGGAAGGTGGCAT
GCATCCAGGCTAGCGTGAATTTACTTTAAATCTTGCATTTAAATAAATTTTCTTTTTATAGCTTTATGAC
TTAGTTTCAATTTATATACTATTTTAATGACATTTTCGATTCATTGATTGAAAGCTTTGTGTTTTTTCTT
GATGCGCTATTGCATTGTTCTTGTCTTTTTCGCCACATGTAATATCTGTAGTAGATACCTGATACATTGT
GGATGCTGAGTGAAATTTTAGTTAATAATGGAGGCGCTCTTAATAATTTTGGGGATATTGGCTTTTTTTT
TTAAAGTTTACAAATGAATTTTTTCCGCCAGGATGCGGCCGCTCTAGACTAGTCCTAGGGGATCCGTCAC
CCGGCCAGCGGAATTCAGGGATGATCTTGAGAAGTTCTTAGAGTCTTACGAGGGAACAGAAGATTTGGAA
CCAGCCAAAGCTGCTATAGCGGAAGCAGATATTTTGCTAAGTAAATGATTAAATAATTAAATATGTGGAA
ATACATTAATCTTTTTATATTTTTGCAGTTCGTTGTCGCTATAATTTATAGTCATCTCGTTAGTTCAAAC
AAGACTTCTTGAAGTGAAAACCAACTTTCAGTCTTCAAACTAAAAATGAAAATCAGTGGAAGAAGGTAAA
CGACTTCATGTTATATATGAATTGAATAGTAATGGAAATAACCAAAAACAGCTCAACAGAAAACAAACAA
AATACGTTAAGACCTGAACTCCTAGCAGAACCATAACTGCCAAATATTTATTATCTGTGGAGATCTTATA
TTCTAAAACCAAAAAAAATATAACTTAAAAGTTAAAAAGAAGATGTTCTAACTGAGGTTCGAACTCAGGA

CCTTTGCCGTGTGAAGGCAACGTGATAGCCACTACACTATTAGAACTACCTTATGGGAAAAAGAAAAATA
GAGTACAACTAGAATGGTAAGATCTGTGACCTTTTCTAAACACTTAATTCCATATAGACAGTTCCCACCC
ACCATAAGGTCACAATTATAATGTCTTTAGAAGACCACTGTCGTTCATCATCTTCCTAAGCCCTCTCTCT
AAAGCGGCATATTTCCGTAATTTGTTCTTCTTTGCACAGGCACGTGAGATGACTCCGATTATTCCCACAT
GCATATTTAGCCTCTCTAGGGGCTCGAGCTATAGCAAGTTCAAGGAAAGAAAACTATTATGATCTGGTCA
CGTGTATAAAATTTATTAATTTTAAAACTATATAATTTATTATTTTTTTATTTTAAAGTTTAAAGTAATT
TTAATAGTATTCTATATTTTAAATAAACATACTTTAAAATTTTATTTAATAATTTATTATTTTTAAATAC
AATGTTTTTATTTAAAACAAAATTATAAGTTAAAAAGTTGTTCCGAAAGTAAAATATATTTTATAGGACG
TCGACATGGAGGCCCAGAATACCCTCCTTGACAGTCTTGACGTGCGCAGCTCAGGGGCATGATGTGACTG
TCGCCCGTACATTTAGCCCATACATCCCCATGTATAATCATTTGCATCCATACATTTTGATGGCCGCACG
GCGCGAAGCAAAAATTACGGCTCCTCGCTGCAGACCTGCGAGCAGGGAAACGCTCCCCTCACAGACGCGT
TGAATTGTCCCCACGCCGCGCCCCTGTAGAGAAATATAAAAGGTTAGGATTTGCCACTGAGGTTCTTCTT
TCATATACTTCCTTTTAAAATCTTGCTAGGATACAGTTCTCACATCACATCCGAACATAAACAACCATGG
GTAAGGAAAAGACTCACGTTTCGAGGCCGCGATTAAATTCCAACATGGATGCTGATTTATATGGGTATAA
ATGGGCTCGCGATAATGTCGGGCAATCAGGTGCGACAATCTATCGATTGTATGGGAAGCCCGATGCGCCA
GAGTTGTTTCTGAAACATGGCAAAGGTAGCGTTGCCAATGATGTTACAGATGAGATGGTCAGACTAAACT
GGCTGACGGAATTTATGCCTCTTCCGACCATCAAGCATTTTATCCGTACTCCTGATGATGCATGGTTACT
CACCACTGCGATCCCCGGCAAAACAGCATTCCAGGTATTAGAAGAATATCCTGATTCAGGTGAAAATATT
GTTGATGCGCTGGCAGTGTTCCTGCGCCGGTTGCATTCGATTCCTGTTTGTAATTGTCCTTTTAACAGCG
ATCGCGTATTTCGTCTCGCTCAGGCGCAATCACGAATGAATAACGGTTTGGTTGATGCGAGTGATTTTGA
TGACGAGCGTAATGGCTGGCCTGTTGAACAAGTCTGGAAAGAAATGCATAAGCTTTTGCCATTCTCACCG
GATTCAGTCGTCACTCATGGTGATTTCTCACTTGATAACCTTATTTTTGACGAGGGGAAATTAATAGGTT
GTATTGATGTTGGACGAGTCGGAATCGCAGACCGATACCAGGATCTTGCCATCCTATGGAACTGCCTCGG
TGAGTTTTCTCCTTCATTACAGAAACGGCTTTTTCAAAAATATGGTATTGATAATCCTGATATGAATAAA
TTGCAGTTTCATTTGATGCTCGATGAGTTTTTCTAATCAGTACTGACAATAAAAAGATTCTTGTTTTCAA
GAACTTGTCATTTGTATAGTTTTTTTATATTGTAGTTGTTCTATTTTAATCAAATGTTAGCGTGATTTAT
ATTTTTTTTCGCCTCGACATCATCTGCCCAGATGCGAAGTTAAGTGCGCAGAAAGTAATATCATGCGTCA
ATCGTATGTGAATGCTGGTCGCTATACTGCCTAGGCCGGCCATAGGGATAACAGGGTAATGTTAACCGGT
TTTTCCATAGGCTCCGCCCCCCTGACGAGCATCACAAAAATCGACGCTCAAGTCAGAGGTGGCGAAACCC
GACAGGACTATAAAGATACCAGGCGTTTCCCCCTGGAAGCTCCCTCGTGCGCTCTCCTGTTCCGACCCTG
CCGCTTACCGGATACCTGTCCGCCTTTCTCCCTTCGGGAAGCGTGGCGCTTTCTCATAGCTCACGCTGTA
GGTATCTCAGTTCGGTGTAGGTCGTTCGCTCCAAGCTGGGCTGTGTGCACGAACCCCCCGTTCAGCCCGA

CCGCTGCGCCTTATCCGGTAACTATCGTCTTGAGTCCAACCCGGTAAGACACGACTTATCGCCACTGGCA
GCAGCCACTGGTAACAGGATTAGCAGAGCGAGGTATGTAGGCGGTGCTACAGAGTTCTTGAAGTGGTGGC
CTAACTACGGCTACACTAGAAGGACAGTATTTGGTATCTGCGCTCTGCTGAAGCCAGTTACCTTCGGAAA
AAGAGTTGGTAGCTCTTGATCCGGCAAACAAACCACCGCTGGTAGCGGTGGTTTTTTTGTTTGCAAGCAG
CAGATTACGCGCAGAAAAAAAGGATCTCAAGAAGATCCTTTGATCTTTTCTACGGGGTCTGACGCTCAGT
GGAACGAAAACTCACGTTAAGGGATTTTGGTCATGAGATTATCAAAAAGGATCTTCACCTAGATCCTTTT
AAATT

Supplementary Figure S3 - Sequence of Cas9_Base plasmid.

Light green: ScP$_{PDC1}$, Red: Cas9 protein, Gray: ScT$_{TDH3}$, Cyan: KmARS7, Green: KmCEN-D, Yellow: *kanMX*

AAAAATGAAGTTTTAAATCAATCTAAAGTATATATGAGTAAACTTGGTCTGACAGTTACCAATGCTTAAT
CAGTGAGGCACCTATCTCAGCGATCTGTCTATTTCGTTCATCCATAGTTGCCTGACTCCCCGTCGTGTAG
ATAACTACGATACGGGAGGGCTTACCATCTGGCCCCAGTGCTGCAATGATACCGCGAGAACCACGCTCAC
CGGCTCCAGATTTATCAGCAATAAACCAGCCAGCCGGAAGGGCCGAGCGCAGAAGTGGTCCTGCAACTTT
ATCCGCCTCCATCCAGTCTATTAATTGTTGCCGGGAAGCTAGAGTAAGTAGTTCGCCAGTTAATAGTTTG
CGCAACGTTGTTGCCATTGCTACAGGCATCGTGGTGTCACGCTCGTCGTTTGGTATGGCTTCATTCAGCT
CCGGTTCCCAACGATCAAGGCGAGTTACATGATCCCCCATGTTGTGCAAAAAAGCGGTTAGCTCCTTCGG
TCCTCCGATCGTTGTCAGAAGTAAGTTGGCCGCAGTGTTATCACTCATGGTTATGGCAGCACTGCATAAT
TCTCTTACTGTCATGCCATCCGTAAGATGCTTTTCTGTGACTGGTGAGTACTCAACCAAGTCATTCTGAG
AATAGTGTATGCGGCGACCGAGTTGCTCTTGCCCGGCGTCAATACGGGATAATACCGCGCCACATAGCAG
AACTTTAAAAGTGCTCATCATTGGAAAACGTTCTTCGGGGCGAAAACTCTCAAGGATCTTACCGCTGTTG
AGATCCAGTTCGATGTAACCCACTCGTGCACCCAACTGATCTTCAGCATCTTTTACTTTCACCAGCGTTT
CTGGGTGAGCAAAAACAGGAAGGCAAAATGCCGCAAAAAAGGGAATAAGGGCGACACGGAAATGTTGAAT
ACTCATACTCTTCCTTTTTCAATATTATTGAAGCATTTATCAGGGTTATTGTCTCATGAGCGGATACATA
TTTGAATGTATTTAGAAAAATAAACAAATAGGGGTTCCGCGTTAATTAATTGAGATAAGCACACTGCACC
CATACCTTCCTTAAAAACGTAGCTTCCAGTTTTTGGTGGTTCCGGCTTCCTTCCCGATTCCGCCCGCTAA
ACGCATATTTTTGTTGCCTGGTGGCATTTGCAAAATGCATAACCTATGCATTTAAAAGATTATGTATGCT
CTTCTGACTTTTCGTGTGATGAGGCTCGTGGAAAAAATGAATAATTTATGAATTTGAGAACAATTTTGTG
TTGTTACGGTATTTTACTATGGAATAATCAATCAATTGAGGATTTTATGCAAATATCGTTTGAATATTTT
TCCGACCCTTTGAGTACTTTTCTTCATAATTGCATAATATTGTCCGCTGCCCCTTTTTCTGTTAGACGGT
GTCTTGATCTACTTGCTATCGTTCAACACCACCTTATTTTCTAACTATTTTTTTTTTAGCTCATTTGAAT
CAGCTTATGGTGATGGCACATTTTTGCATAAACCTAGCTGTCCTCGTTGAACATAGGAAAAAAAAATATA
TAAACAAGGCTCTTTCACTCTCCTTGCAATCAGATTTGGGTTTGTTCCCTTTATTTTCATATTTCTTGTC
ATATTCCTTTCTCAATTATTATTTTCTACTCATAACCTCACGCAAAATAACACAGTCAAATCAATCAAAG
CTAGCGAAATGGCACCGAAGAAGAAGCGTAAAGTCGGAATCCACGGAGTTCCTGCGGCAATGGACAAGAA
GTACTCCATTGGGCTCGCTATCGGCACAAACAGCGTCGGTTGGGCCGTCATTACGGACGAGTACAAGGTG
CCGAGCAAAAAATTCAAAGTTCTGGGCAATACCGATCGCCACAGCATAAAGAAGAACCTCATTGGCGCCC
TCCTGTTCGACTCCGGGGAGACGGCCGAAGCCACGCGGCTCAAAAGAACAGCACGGCGCAGATATACCCG
CAGAAAGAATCGGATCTGCTACCTGCAGGAGATCTTTAGTAATGAGATGGCTAAGGTGGATGACTCTTTC
TTCCATAGGCTGGAGGAGTCCTTTTTGGTGGAGGAGGATAAAAAGCACGAGCGCCACCCAATCTTTGGCA
ATATCGTGGACGAGGTGGCGTACCATGAAAAGTACCCAACCATATATCATCTGAGGAAGAAGCTTGTAGA
CAGTACTGATAAGGCTGACTTGCGGTTGATCTATCTCGCGCTGGCGCATATGATCAAATTTCGGGGACAC

TTCCTCATCGAGGGGGACCTGAACCCAGACAACAGCGATGTCGACAAACTCTTTATCCAACTGGTTCAGA
CTTACAATCAGCTTTTCGAAGAGAACCCGATCAACGCATCCGGAGTTGACGCCAAAGCAATCCTGAGCGC
TAGGCTGTCCAAATCCCGGCGGCTCGAAAACCTCATCGCACAGCTCCCTGGGGAGAAGAAGAACGGCCTG
TTTGGTAATCTTATCGCCCTGTCACTCGGGCTGACCCCCAACTTTAAATCTAACTTCGACCTGGCCGAAG
ATGCCAAGCTTCAACTGAGCAAAGACACCTACGATGATGATCTCGACAATCTGCTGGCCCAGATCGGCGA
CCAGTACGCAGACCTTTTTTTGGCGGCAAAGAACCTGTCAGACGCCATTCTGCTGAGTGATATTCTGCGA
GTGAACACGGAGATCACCAAAGCTCCGCTGAGCGCTAGTATGATCAAGCGCTATGATGAGCACCACCAAG
ACTTGACTTTGCTGAAGGCCCTTGTCAGACAGCAACTGCCTGAGAAGTACAAGGAAATTTTCTTCGATCA
GTCTAAAAATGGCTACGCCGGATACATTGACGGCGGAGCAAGCCAGGAGGAATTTTACAAATTTATTAAG
CCCATCTTGGAAAAAATGGACGGCACCGAGGAGCTGCTGGTAAAGCTTAACAGAGAAGATCTGTTGCGCA
AACAGCGCACTTTCGACAATGGAAGCATCCCCCACCAGATTCACCTGGGCGAACTGCACGCTATCCTCAG
GCGGCAAGAGGATTTCTACCCCTTTTTGAAAGATAACAGGGAAAAGATTGAGAAAATCCTCACATTTCGG
ATACCCTACTATGTAGGCCCCCTCGCCCGGGGAAATTCCAGATTCGCGTGGATGACTCGCAAATCAGAAG
AGACCATCACTCCCTGGAACTTCGAGGAAGTCGTGGATAAGGGGGCCTCTGCCCAGTCCTTCATCGAAAG
GATGACTAACTTTGATAAAAATCTGCCTAACGAAAAGGTGCTTCCTAAACACTCTCTGCTGTACGAGTAC
TTCACAGTTTATAACGAGCTCACCAAGGTCAAATACGTCACAGAAGGGATGAGAAAGCCAGCATTCCTGT
CTGGAGAGCAGAAGAAAGCTATCGTGGACCTCCTCTTCAAGACGAACCGGAAAGTTACCGTGAAACAGCT
CAAAGAAGACTATTTCAAAAAGATTGAATGTTTCGACTCTGTTGAAATCAGCGGAGTGGAGGATCGCTTC
AACGCATCCCTGGGAACGTATCACGATCTCCTGAAAATCATTAAAGACAAGGACTTCCTGGACAATGAGG
AGAACGAGGACATTCTTGAGGACATTGTCCTCACCCTTACGTTGTTTGAAGATAGGGAGATGATTGAAGA
ACGCTTGAAAACTTACGCTCATCTCTTCGACGACAAAGTCATGAAACAGCTCAAGAGGCGCCGATATACA
GGATGGGGGCGGCTGTCAAGAAAACTGATCAATGGGATCCGAGACAAGCAGAGTGGAAAGACAATCCTGG
ATTTTCTTAAGTCCGATGGATTTGCCAACCGGAACTTCATGCAGTTGATCCATGATGACTCTCTCACCTT
TAAGGAGGACATCCAGAAAGCACAAGTTTCTGGCCAGGGGGACAGTCTTCACGAGCACATCGCTAATCTT
GCAGGTAGCCCAGCTATCAAAAAGGGAATACTGCAGACCGTTAAGGTCGTGGATGAACTCGTCAAAGTAA
TGGGAAGGCATAAGCCCGAGAATATCGTTATCGAGATGGCCCGAGAGAACCAAACTACCCAGAAGGGACA
GAAGAACAGTAGGGAAAGGATGAAGAGGATTGAAGAGGGTATAAAAGAACTGGGGTCCCAAATCCTTAAG
GAACACCCAGTTGAAAACACCCAGCTTCAGAATGAGAAGCTCTACCTGTACTACCTGCAGAACGGCAGGG
ACATGTACGTGGATCAGGAACTGGACATCAATCGGCTCTCCGACTACGACGTGGATCATATCGTGCCCCA
GTCTTTTCTCAAAGATGATTCTATTGATAATAAAGTGTTGACAAGATCCGATAAAAATAGAGGGAAGAGT
GATAACGTCCCCTCAGAAGAAGTTGTCAAGAAAATGAAAAATTATTGGCGGCAGCTGCTGAACGCCAAAC
TGATCACACAACGGAAGTTCGATAATCTGACTAAGGCTGAACGAGGTGGCCTGTCTGAGTTGGATAAAGC

CGGCTTCATCAAAAGGCAGCTTGTTGAGACACGCCAGATCACCAAGCACGTGGCCCAAATTCTCGATTCA
CGCATGAACACCAAGTACGATGAAAATGACAAACTGATTCGAGAGGTGAAAGTTATTACTCTGAAGTCTA
AGCTGGTCTCAGATTTCAGAAAGGACTTTCAGTTTTATAAGGTGAGAGAGATCAACAATTACCACCATGC
GCATGATGCCTACCTGAATGCAGTGGTAGGCACTGCACTTATCAAAAAATATCCCAAGCTTGAATCTGAA
TTTGTTTACGGAGACTATAAAGTGTACGATGTTAGGAAAATGATCGCAAAGTCTGAGCAGGAAATAGGCA
AGGCCACCGCTAAGTACTTCTTTTACAGCAATATTATGAATTTTTTCAAGACCGAGATTACACTGGCCAA
TGGAGAGATTCGGAAGCGACCACTTATCGAAACAAACGGAGAAACAGGAGAAATCGTGTGGGACAAGGGT
AGGGATTTCGCGACAGTCCGGAAGGTCCTGTCCATGCCGCAGGTGAACATCGTTAAAAAGACCGAAGTAC
AGACCGGAGGCTTCTCCAAGGAAAGTATCCTCCCGAAAAGGAACAGCGACAAGCTGATCGCACGCAAAAA
AGATTGGGACCCCAAGAAATACGGCGGATTCGATTCTCCTACAGTCGCTTACAGTGTACTGGTTGTGGCC
AAAGTGGAGAAAGGGAAGTCTAAAAAACTCAAAAGCGTCAAGGAACTGCTGGGCATCACAATCATGGAGC
GATCAAGCTTCGAAAAAAACCCCATCGACTTTCTCGAGGCGAAAGGATATAAAGAGGTCAAAAAAGACCT
CATCATTAAGCTTCCCAAGTACTCTCTCTTTGAGCTTGAAAACGGCCGGAAACGAATGCTCGCTAGTGCG
GGCGAGCTGCAGAAAGGTAACGAGCTGGCACTGCCCTCTAAATACGTTAATTTCTTGTATCTGGCCAGCC
ACTATGAAAAGCTCAAAGGGTCTCCCGAAGATAATGAGCAGAAGCAGCTGTTCGTGGAACAACACAAACA
CTACCTTGATGAGATCATCGAGCAAATAAGCGAATTCTCCAAAAGAGTGATCCTCGCCGACGCTAACCTC
GATAAGGTGCTTTCTGCTTACAATAAGCACAGGGATAAGCCCATCAGGGAGCAGGCAGAAAACATTATCC
ACTTGTTTACTCTGACCAACTTGGGCGCGCCTGCAGCCTTCAAGTACTTCGACACCACCATAGACAGAAA
GCGGTACACCTCTACAAAGGAGGTCCTGGACGCCACACTGATTCATCAGTCAATTACGGGGCTCTATGAA
ACAAGAATCGACCTCTCTCAGCTCGGTGGAGACAGCAGGGCTGACCCCAAGAAGAAGAGGAAGGTGGGTG
GAGGAGGTACCGGCGGTGGAGGCTCAGCAGAATACGTACGAGCTCTGTTTGACTTCAATGGGAATGACGA
GGAGGATCTCCCCTTTAAGAAGGGCGATATTCTCCGCATCAGAGATAAGCCCGAAGAACAATGGTGGAAT
GCCGAGGATAGCGAAGGGAAAAGGGGCATGATTCTGGTGCCATATGTGGAGAAATATTCCGGTGACTACA
AAGACCATGATGGGGATTACAAAGACCACGACATCGACTACAAAGACGACGACGATAAATCAGGGATGAC
AGACGCCGAGTACGTGCGCATTCATGAGAAACTGGATATTTACACCTTCAAGAAGCAGTTCTTCAACAAC
AAGAAATCTGTGTCACACCGCTGCTACGTGCTGTTTGAGTTGAAGCGAAGGGGCGAAAGAAGGGCTTGCT
TTTGGGGCTATGCCGTCAACAAGCCCCAAAGTGGCACCGAGAGAGGAATACACGCTGAGATATTCAGTAT
CCGAAAGGTGGAAGAGTATCTTCGGGATAATCCTGGGCAGTTTACGATCAACTGGTATTCCAGCTGGAGT
CCTTGCGCTGATTGTGCCGAGAAAATTCTGGAATGGTATAATCAGGAACTTCGGGGAAACGGGCACACAT
TGAAAATCTGGGCCTGCAAGCTGTACTACGAGAAGAATGCCCGGAACCAGATAGGACTCTGGAATCTGAG
GGACAATGGTGTAGGCCTGAACGTGATGGTTTCCGAGCACTATCAGTGTTGTCGGAAGATTTTCATCCAA
AGCTCTCATAACCAGCTCAATGAAAACCGCTGGTTGGAGAAAACACTGAAACGTGCGGAGAAGCGGAGAT

CCGAGCTGAGCATCATGATCCAGGTCAAGATTCTGCATACCACTAAGTCTCCAGCCGTTGGTCCCAAGAA
GAAAAGAAAAGTCGCATGCATCCAGGCTAGCGTGAATTTACTTTAAATCTTGCATTTAAATAAATTTTCT
TTTTATAGCTTTATGACTTAGTTTCAATTTATATACTATTTTAATGACATTTTCGATTCATTGATTGAAA
GCTTTGTGTTTTTTCTTGATGCGCTATTGCATTGTTCTTGTCTTTTTCGCCACATGTAATATCTGTAGTA
GATACCTGATACATTGTGGATGCTGAGTGAAATTTTAGTTAATAATGGAGGCGCTCTTAATAATTTTGGG
GATATTGGCTTTTTTTTTTAAAGTTTACAAATGAATTTTTTCCGCCAGGATGCGGCCGCTCTAGACTAGT
CCTAGGGGATCCGTCACCCGGCCAGCGGAATTCAGGGATGATCTTGAGAAGTTCTTAGAGTCTTACGAGG
GAACAGAAGATTTGGAACCAGCCAAAGCTGCTATAGCGGAAGCAGATATTTTGCTAAGTAAATGATTAAA
TAATTAAATATGTGGAAATACATTAATCTTTTTATATTTTTGCAGTTCGTTGTCGCTATAATTTATAGTC
ATCTCGTTAGTTCAAACAAGACTTCTTGAAGTGAAAACCAACTTTCAGTCTTCAAACTAAAAATGAAAAT
CAGTGGAAGAAGGTAAACGACTTCATGTTATATATGAATTGAATAGTAATGGAAATAACCAAAAACAGCT
CAACAGAAAACAAACAAAATACGTTAAGACCTGAACTCCTAGCAGAACCATAACTGCCAAATATTTATTA
TCTGTGGAGATCTTATATTCTAAAACCAAAAAAAATATAACTTAAAAGTTAAAAAGAAGATGTTCTAACT
GAGGTTCGAACTCAGGACCTTTGCCGTGTGAAGGCAACGTGATAGCCACTACACTATTAGAACTACCTTA
TGGGAAAAAGAAAAATAGAGTACAACTAGAATGGTAAGATCTGTGACCTTTTCTAAACACTTAATTCCAT
ATAGACAGTTCCCACCCACCATAAGGTCACAATTATAATGTCTTTAGAAGACCACTGTCGTTCATCATCT
TCCTAAGCCCTCTCTCTAAAGCGGCATATTTCCGTAATTTGTTCTTCTTTGCACAGGCACGTGAGATGAC
TCCGATTATTCCCACATGCATATTTAGCCTCTCTAGGGGCTCGAGCTATAGCAAGTTCAAGGAAAGAAAA
CTATTATGATCTGGTCACGTGTATAAAATTTATTAATTTTAAAACTATATAATTTATTATTTTTTTATTT
TAAAGTTTAAAGTAATTTTAATAGTATTCTATATTTTAAATAAACATACTTTAAAATTTTATTTAATAAT
TTATTATTTTTAAATACAATGTTTTTATTTAAAACAAAATTATAAGTTAAAAAGTTGTTCCGAAAGTAAA
ATATATTTTATAGGACGTCGACATGGAGGCCCAGAATACCCTCCTTGACAGTCTTGACGTGCGCAGCTCA
GGGGCATGATGTGACTGTCGCCCGTACATTTAGCCCATACATCCCCATGTATAATCATTTGCATCCATAC
ATTTTGATGGCCGCACGGCGCGAAGCAAAAATTACGGCTCCTCGCTGCAGACCTGCGAGCAGGGAAACGC
TCCCCTCACAGACGCGTTGAATTGTCCCCACGCCGCGCCCCTGTAGAGAAATATAAAAGGTTAGGATTTG
CCACTGAGGTTCTTCTTTCATATACTTCCTTTTAAAATCTTGCTAGGATACAGTTCTCACATCACATCCG
AACATAAACAACCATGGGTAAGGAAAAGACTCACGTTTCGAGGCCGCGATTAAATTCCAACATGGATGCT
GATTTATATGGGTATAAATGGGCTCGCGATAATGTCGGGCAATCAGGTGCGACAATCTATCGATTGTATG
GGAAGCCCGATGCGCCAGAGTTGTTTCTGAAACATGGCAAAGGTAGCGTTGCCAATGATGTTACAGATGA
GATGGTCAGACTAAACTGGCTGACGGAATTTATGCCTCTTCCGACCATCAAGCATTTTATCCGTACTCCT
GATGATGCATGGTTACTCACCACTGCGATCCCCGGCAAAACAGCATTCCAGGTATTAGAAGAATATCCTG
ATTCAGGTGAAAATATTGTTGATGCGCTGGCAGTGTTCCTGCGCCGGTTGCATTCGATTCCTGTTTGTAA

TTGTCCTTTTAACAGCGATCGCGTATTTCGTCTCGCTCAGGCGCAATCACGAATGAATAACGGTTTGGTT
GATGCGAGTGATTTTGATGACGAGCGTAATGGCTGGCCTGTTGAACAAGTCTGGAAAGAAATGCATAAGC
TTTTGCCATTCTCACCGGATTCAGTCGTCACTCATGGTGATTTCTCACTTGATAACCTTATTTTTGACGA
GGGGAAATTAATAGGTTGTATTGATGTTGGACGAGTCGGAATCGCAGACCGATACCAGGATCTTGCCATC
CTATGGAACTGCCTCGGTGAGTTTTCTCCTTCATTACAGAAACGGCTTTTTCAAAAATATGGTATTGATA
ATCCTGATATGAATAAATTGCAGTTTCATTTGATGCTCGATGAGTTTTTCTAATCAGTACTGACAATAAA
AAGATTCTTGTTTTCAAGAACTTGTCATTTGTATAGTTTTTTTATATTGTAGTTGTTCTATTTTAATCAA
ATGTTAGCGTGATTTATATTTTTTTTCGCCTCGACATCATCTGCCCAGATGCGAAGTTAAGTGCGCAGAA
AGTAATATCATGCGTCAATCGTATGTGAATGCTGGTCGCTATACTGCCTAGGCCGGCCATAGGGATAACA
GGGTAATGTTAACCGGTTTTTCCATAGGCTCCGCCCCCCTGACGAGCATCACAAAAATCGACGCTCAAGT
CAGAGGTGGCGAAACCCGACAGGACTATAAAGATACCAGGCGTTTCCCCCTGGAAGCTCCCTCGTGCGCT
CTCCTGTTCCGACCCTGCCGCTTACCGGATACCTGTCCGCCTTTCTCCCTTCGGGAAGCGTGGCGCTTTC
TCATAGCTCACGCTGTAGGTATCTCAGTTCGGTGTAGGTCGTTCGCTCCAAGCTGGGCTGTGTGCACGAA
CCCCCCGTTCAGCCCGACCGCTGCGCCTTATCCGGTAACTATCGTCTTGAGTCCAACCCGGTAAGACACG
ACTTATCGCCACTGGCAGCAGCCACTGGTAACAGGATTAGCAGAGCGAGGTATGTAGGCGGTGCTACAGA
GTTCTTGAAGTGGTGGCCTAACTACGGCTACACTAGAAGGACAGTATTTGGTATCTGCGCTCTGCTGAAG
CCAGTTACCTTCGGAAAAAGAGTTGGTAGCTCTTGATCCGGCAAACAAACCACCGCTGGTAGCGGTGGTT
TTTTTGTTTGCAAGCAGCAGATTACGCGCAGAAAAAAAGGATCTCAAGAAGATCCTTTGATCTTTTCTAC
GGGGTCTGACGCTCAGTGGAACGAAAACTCACGTTAAGGGATTTTGGTCATGAGATTATCAAAAAGGATC
TTCACCTAGATCCTTTTAAATT

Supplementary Figure S4 - Plasmid nCas9-CDA_Base sequences.

Light green: ScP_{PDC1}, Red: nCas9 protein, Gray: ScT_{TDH3}, Cyan: KmARS7, Green: KmCEN-D, Yellow: *kanMX*

AGCGCCCAATACGCAAACCGCCTCTCCCCGCGCGTTGGCCGATTCATTAATGCAGCTGGCACGACAGGTT
TCCCGACTGGAAAGCGGGCAGTGAGCGCAACGCAATTAATGTGAGTTAGCTCACTCATTAGGCACCCCAG
GCTTTACACTTTATGCTTCCGGCTCGTATGTTGTGTGGAATTGTGAGCGGATAACAATTTCACACAGGAA
ACAGCTATGACCATGATTACGCCAAGCTCAGAATTAACCCTCACTAAAGGGACTAGTCCTGCAGGTTTAA
ACGAATTCGCCCTTCAGAGAGTCTGCAACACCGATTTATCGGTTTGCAGGTAACACAATACCGATTACTT
GGTGTCTGAGGAGATCTCAATTCTCTCTTTTTCCTTCAACTTTTATTTTCTTGGTATGCTTCTTGTGCAT
TCGCCTATTAATTTATATTAGCGATTCTTATGACATCGGCCATCACTGAGTATAGTGAGTAGTAGTGGTT
GATATTTGAAATCTATTATAGATAAGTTCCTTTACATATCGTGCAGCACTTGTTTATGTGAACTATTAGT
AGCTATATTGGCTATATACAGAGAGAAAGCTATAACTATTTATATCATAACTGTTTGTATAACTTAACGA
CCCATGCTTTGGTTCAAAGCAGCGTTTTGCTCTTGGAAACGTTGTCCAACACGTTCACTATGTTTCAAGA
ACTTTTCTGAGCATTTCATTATGCAGCTTTGCTCCTTGGAAGTTAGCTTAGCAGAGGTAAAGTCGTTGAC
ACAGTCACTGAAACATCTTTCGACCAAGTTGGAGTATAGACGCATGAAGTCCTTCATTTGCTTTTGTTCC
ACAATTCTTTGGAACTCTTGTTGTTCCTTACCATTTAGTTGATCCATTTTTAACTGTGTATTTGATTTTT
TTGTTCTAGCAATCGACGGTCCAAGACTCAATAAAAATTGACTCTTGTATCTTTCTTCACACTAGTAGTA
AAGCAGAAAGTCTAGCAGAAATGTGTATCTTTTATAACGGTCAATGGTCTAAGAAGCAAGGTTTGGTTCT
TCGCACTTGTATTAAACTCTTTTTCTTTGGTTGTGAAATTTCACATAATGTACTTCACCAAAAAACAAAA
AAAAAAAAAAAAAAAAAAATTTTCGGTGCAAAAAACAGCTTCTAGCACGTGACTAAACTTGTAGCGTATA
CAATGTGCGCAATGCCATTACACTCTAACATCTCTGTATAGTTTATATATAAACGATCAACTTGTAACTT
CTGATCCGAGTACACTCGAACCTCTGCTTGTTCTCAGTATAGCGACCAGCATTCACATACGATTGACGCA
TGATATTACTTTCTGCGCACTTAACTTCGCATCTGGGCAGATGATGTCGAGGCGAAAAAAAATATAAATC
ACGCTAACATTTGATTAAAATAGAACAACTACAATATAAAAAAACTATACAAATGACAAGTTCTTGAAAA
CAAGAATCTTTTTATTGTCAGTACTGATCATGAGATGCCTGCAAGCAATTCGTTCTGTATCAGGCGCAGG
AGCGTCCCGTCCGGGTCGACCAAAGCGGCCATCGTGCCTCCCCACTCCTGCAGTTCGGGGGCATGGATGC
GCGGATAGCCGCTGCTGGTTTCCTGGATGCCGACGGATTTGCACTGCCGGTAGAACTCCGCGAGGTCGTC
CAGCCTCAGGCAGCAGCTGAACCAACTCGCGAGGGGATCGAGCCCGGGGTGGGCGAAGAACTCCAGCATG
AGATCCCCGCGCTGGAGGATCATCCAGCCGGCGTCCCGGAAAACGATTCCGAAGCCCAACCTTTCATAGA
AGGCGGCGGTGGGATCGAAATCTCGTGATGGCAGGTTGGGCGTCGCTTGGTCGGTCATACCCATGCCGGT
ACCGGATCCCTTGTACAGCTCGTCCATGCCGAGAGTGATCCCGGCGGCGGTCACGAACTCCAGCAGGACC
ATGTGATCGCGCTTCTCGTTGGGGTCTTTGCTCAGGGCGGACTGGGTGCTCAGGTAGTGGTTGTCGGGCA
GCAGCACGGGGCCGTCGCCGATGGGGGTGTTCTGCTGGTAGTGGTCGGCGAGCTGCACGCTGCCGTCCTC
GATGTTGTGGCGGATCTTGAAGTTCACCTTGATGCCGTTCTTCTGCTTGTCGGCCATGATATAGACGTTG
TGGCTGTTGTAGTTGTACTCCAGCTTGTGCCCCAGGATGTTGCCGTCCTCCTTGAAGTCGATGCCCTTCA

GCTCGATGCGGTTCACCAGGGTGTCGCCCTCGAACTTCACCTCGGCGCGGGTCTTGTAGTTGCCGTCGTC
CTTGAAGAAGATGGTGCGCTCCTGGACGTAGCCTTCGGGCATGGCGGACTTGAAGAAGTCGTGCTGCTTC
ATGTGGTCGGGGTAGCGGCTGAAGCACTGCACGCCGTAGGTCAGGGTGGTCACGAGGGTGGGCCAGGGCA
CGGGCAGCTTGCCGGTGGTGCAGATGAACTTCAGGGTCAGCTTGCCGTAGGTGGCATCGCCCTCGCCCTC
GCCGGACACGCTGAACTTGTGGCCGTTTACGTCGCCGTCCAGCTCGACCAGGATGGGCACCACCCCGGTG
AACAGCTCCTCGCCCTTGCTCACCATGCTAGCTTTGATTGATTTGACTGTGTTATTTTGCGTGAGGTTAT
GAGTAGAAAATAATAATTGAGAAAGGAATATGACAAGAAATATGAAAATAAAGGGAACAAACCCAAATCT
GATTGCAAGGAGAGTGAAAGAGCCTTGTTTATATATTTTTTTTTCCTATGTTCAACGAGGACAGCTAGGT
TTATGCAAAAATGTGCCATCACCATAAGCTGATTCAAATGAGCTAAAAAAAAAATAGTTAGAAAATAAGG
TGGTGTTGAACGATAGCAAGTAGATCAAGACACCGTCTAACAGAAAAAGGGGCAGCGGACAATATTATGC
AATTATGAAGAAAAGTACTCAAAGGGTCGGAAAAATATTCAAACGATATTTGCATAAAATCCTCAATTGA
TTGATTATTCCATAGTAAAATACCGTAACAACACAAAATTGTTCTCAAATTCATAAATTATTCATTTTTT
CCACGAGCCTCATCACACGAAAAGTCAGAAGAGCATACATAATCTTTTAAATGCATAGGTTATGCATTTT
GCAAATGCCACCAGGCAACAAAAATATGCGTTTAGCGGGCGGAATCGGGAAGGAAGCCGGAACCACCAAA
AACTGGAAGCTACGTTTTTAAGGAAGGTATGGGTGCAGTGTGCTTATCTCAACCTTTGATTAGTTTATGG
GCTAGCTTATGATACTTCCTCTTGCTGTTCCAGTTTACAAATGATTTAAACGAACTTCTACACTTTTACG
GTAGTATTTCTTGTTGGTGATGAGCTCAGATTCAATATGACTGCTTAATCAGTGAAAATTAATGGAAACA
GCCAGACACACAGCCTTTGAAAAATGTTAAAAACGATCACATATATGTAGTCACGTGGTTAGTGCATAGA
GTCAGGCGATGGTGGTTAGTTCCCTAGCCAGAGCCATTCCCCTCTTGTTTCGAAATGCGATGACTGAATG
GGGAAGAAGCTCTTAGTATTTCTGTAGTTTTTTTGCCGATTTCTATTGTTGCCTGCCCCGGACTTTCTT
TTACCTTTTGTAGCTACAACTATTAATTATAATGCATAATAGATCTAGAAGCGTATTGTGATGAGCATCC
TTGGAAATAAGAAAGGAAGTCACAGGTACAGTAAAAAAAGAGCCGTGACAGGCACCTAATTTCAGTTTTG
ATTGAGGTATAATGAGCTCAGAAGACCAAGATGCTCTAAAAGTAAAGCAGAATGGTCTTTCCAATCAGAA
TTCGTCACTTGTTGGTGCTTTAACTGCTGGTGGAAGATCTATGGTTTATCAGATGACTTCATTCTATATG
AGAACTCCACTAAAACTGTTTCGACCGGCCAGATTTGACTATACGCATTATATTCGAGTACTGTTGACTG
GGAGTGATGATACAAATAAGGATTCTAAGGTACGACGTTCAAAATATAAGTTTTGGAGTCCCAAATATAC
ATACTACTTCGAAAATTCCTCCATTGGAATAGTTACTAAGGCACTAAATAAGTATGGGTGGAAAGTTATT
CCTGATAGAATATTGCCTCCTTTGGTGGCAAATTCCCTTGCTGGGGTAGTTCTTTATACCACATACTTGA
CTACCCTGAATAATTTCCCAGTCAGATCAGTGGAGCCAGCTTTTCAACACAATTTGTTTGACTACTTAAG
GTAAGGGCGAATTCGCGGCCGCTAAATTCAATTCGCCCTATAGTGAGTCGTATTACAATTCACTGGCCGT
CGTTTTACAACGTCGTGACTGGGAAAACCCTGGCGTTACCCAACTTAATCGCCTTGCAGCACATCCCCCT
TTCGCCAGCTGGCGTAATAGCGAAGAGGCCCGCACCGATCGCCCTTCCCAACAGTTGCGCAGCCTATACG

TACGGCAGTTTAAGGTTTACACCTATAAAAGAGAGAGCCGTTATCGTCTGTTTGTGGATGTACAGAGTGA
TATTATTGACACGCCGGGGCGACGGATGGTGATCCCCCTGGCCAGTGCACGTCTGCTGTCAGATAAAGTC
TCCCGTGAACTTTACCCGGTGGTGCATATCGGGGATGAAAGCTGGCGCATGATGACCACCGATATGGCCA
GTGTGCCGGTCTCCGTTATCGGGGAAGAAGTGGCTGATCTCAGCCACCGCGAAAATGACATCAAAAACGC
CATTAACCTGATGTTCTGGGGAATATAAATGTCAGGCATGAGATTATCAAAAAGGATCTTCACCTAGATC
CTTTTCACGTAGAAAGCCAGTCCGCAGAAACGGTGCTGACCCCGGATGAATGTCAGCTACTGGGCTATCT
GGACAAGGGAAAACGCAAGCGCAAAGAGAAAGCAGGTAGCTTGCAGTGGGCTTACATGGCGATAGCTAGA
CTGGGCGGTTTTATGGACAGCAAGCGAACCGGAATTGCCAGCTGGGGCGCCCTCTGGTAAGGTTGGGAAG
CCCTGCAAAGTAAACTGGATGGCTTTCTTGCCGCCAAGGATCTGATGGCGCAGGGGATCAAGCTCTGATC
AAGAGACAGGATGAGGATCGTTTCGCATGATTGAACAAGATGGATTGCACGCAGGTTCTCCGGCCGCTTG
GGTGGAGAGGCTATTCGGCTATGACTGGGCACAACAGACAATCGGCTGCTCTGATGCCGCCGTGTTCCGG
CTGTCAGCGCAGGGGCGCCCGGTTCTTTTTGTCAAGACCGACCTGTCCGGTGCCCTGAATGAACTGCAAG
ACGAGGCAGCGCGGCTATCGTGGCTGGCCACGACGGGCGTTCCTTGCGCAGCTGTGCTCGACGTTGTCAC
TGAAGCGGGAAGGGACTGGCTGCTATTGGGCGAAGTGCCGGGGCAGGATCTCCTGTCATCTCACCTTGCT
CCTGCCGAGAAAGTATCCATCATGGCTGATGCAATGCGGCGGCTGCATACGCTTGATCCGGCTACCTGCC
CATTCGACCACCAAGCGAAACATCGCATCGAGCGAGCACGTACTCGGATGGAAGCCGGTCTTGTCGATCA
GGATGATCTGGACGAAGAGCATCAGGGGCTCGCGCCAGCCGAACTGTTCGCCAGGCTCAAGGCGAGCATG
CCCGACGGCGAGGATCTCGTCGTGACCCATGGCGATGCCTGCTTGCCGAATATCATGGTGGAAAATGGCC
GCTTTTCTGGATTCATCGACTGTGGCCGGCTGGGTGTGGCGGACCGCTATCAGGACATAGCGTTGGCTAC
CCGTGATATTGCTGAAGAGCTTGGCGGCGAATGGGCTGACCGCTTCCTCGTGCTTTACGGTATCGCCGCT
CCCGATTCGCAGCGCATCGCCTTCTATCGCCTTCTTGACGAGTTCTTCTGAATTATTAACGCTTACAATT
TCCTGATGCGGTATTTTCTCCTTACGCATCTGTGCGGTATTTCACACCGCATCAGGTGGCACTTTTCGGG
GAAATGTGCGCGGAACCCCTATTTGTTTATTTTTCTAAATACATTCAAATATGTATCCGCTCATGAGATT
ATCAAAAAGGATCTTCACCTAGATCCTTTTAAATTAAAAATGAAGTTTTAAATCAATCTAAAGTATATAT
GAGTAAACTTGGTCTGACAGTTACCAATGCTTAATCAGTGAGGCACCTATCTCAGCGATCTGTCTATTTC
GTTCATCCATAGTTGCCTGACTCCCCGTCGTGTAGATAACTACGATACGGGAGGGCTTACCATCTGGCCC
CAGTGCTGCAATGATACCGCGAGACCCACGCTCACCGGCTCCAGATTTATCAGCAATAAACCAGCCAGCC
GGAAGGGCCGAGCGCAGAAGTGGTCCTGCAACTTTATCCGCCTCCATCCAGTCTATTAATTGTTGCCGGG
AAGCTAGAGTAAGTAGTTCGCCAGTTAATAGTTTGCGCAACGTTGTTGCCATTGCTACAGGCATCGTGGT
GTCACGCTCGTCGTTTGGTATGGCTTCATTCAGCTCCGGTTCCCAACGATCAAGGCGAGTTACATGATCC
CCCATGTTGTGCAAAAAAGCGGTTAGCTCCTTCGGTCCTCCGATCGTTGTCAGAAGTAAGTTGGCCGCAG
TGTTATCACTCATGGTTATGGCAGCACTGCATAATTCTCTTACTGTCATGCCATCCGTAAGATGCTTTTC

TGTGACTGGTGAGTACTCAACCAAGTCATTCTGAGAATAGTGTATGCGGCGACCGAGTTGCTCTTGCCCG
GCGTCAATACGGGATAATACCGCGCCACATAGCAGAACTTTAAAAGTGCTCATCATTGGAAAACGTTCTT
CGGGGCGAAAACTCTCAAGGATCTTACCGCTGTTGAGATCCAGTTCGATGTAACCCACTCGTGCACCCAA
CTGATCTTCAGCATCTTTTACTTTCACCAGCGTTTCTGGGTGAGCAAAAACAGGAAGGCAAAATGCCGCA
AAAAAGGGAATAAGGGCGACACGGAAATGTTGAATACTCATACTCTTCCTTTTTCAATATTATTGAAGCA
TTTATCAGGGTTATTGTCTCATGACCAAAATCCCTTAACGTGAGTTTTCGTTCCACTGAGCGTCAGACCC
CGTAGAAAAGATCAAAGGATCTTCTTGAGATCCTTTTTTTCTGCGCGTAATCTGCTGCTTGCAAACAAAA
AAACCACCGCTACCAGCGGTGGTTTGTTTGCCGGATCAAGAGCTACCAACTCTTTTTCCGAAGGTAACTG
GCTTCAGCAGAGCGCAGATACCAAATACTGTTCTTCTAGTGTAGCCGTAGTTAGGCCACCACTTCAAGAA
CTCTGTAGCACCGCCTACATACCTCGCTCTGCTAATCCTGTTACCAGTGGCTGCTGCCAGTGGCGATAAG
TCGTGTCTTACCGGGTTGGACTCAAGACGATAGTTACCGGATAAGGCGCAGCGGTCGGGCTGAACGGGGG
GTTCGTGCACACAGCCCAGCTTGGAGCGAACGACCTACACCGAACTGAGATACCTACAGCGTGAGCTATG
AGAAAGCGCCACGCTTCCCGAAGGGAGAAAGGCGGACAGGTATCCGGTAAGCGGCAGGGTCGGAACAGGA
GAGCGCACGAGGGAGCTTCCAGGGGGAAACGCCTGGTATCTTTATAGTCCTGTCGGGTTTCGCCACCTCT
GACTTGAGCGTCGATTTTTGTGATGCTCGTCAGGGGGGCGGAGCCTATGGAAAAACGCCAGCAACGCGGC
CTTTTTACGGTTCCTGGCCTTTTGCTGGCCTTTTGCTCACATGTTCTTTCCTGCGTTATCCCCTGATTCT
GTGGATAACCGTATTACCGCCTTTGAGTGAGCTGATACCGCTCGCCGCAGCCGAACGACCGAGCGCAGCG
AGTCAGTGAGCGAGGAAGCGGAAG

Supplementary Figure S5 - Plasmid TOPO/F7 sequences.
Yellow: bleomycin gene, Cyan: GFP coding sequence, Green: ScP_{PDC1}

Supplementary Table S1 - Primers used in this study

Primer name	Sequence (5'-3')	Purpose
Universal primers for sgRNA cassette construction		
P_Km01_013(pKmSNR52_Fw)	aagctagctctagaTGCCGCAAATAGGGCAGG	amplification of $P_{KmSNR52}$ region
P_Km01_014(tracerRNA_Fw)	GTTTTAGAGCTAGAAATAGCAAG	amplification of tracrRNA_T_{sup4} fragment
P_Km01_015(tracerRNA_Rv)	cctgaattcGCGGCCGCatactagtCATAAAAAACAAAAAAAGCACCACC	amplification of tracrRNA_T_{sup4} fragment
Target-specific primers		
P_Km01_016(single1(NEJ1)_Fw)	CTAGCTCTAAAACCTTATTATCAGTATTTTGTAGATTCGAACTGCGGACGTTG	amplification of target *NEJ1*
P_Km01_017(single2(DNL4)_Fw)	CTAGCTCTAAAACTCTCCAGAGTTTAAATGGTTGATTCGAACTGCGGACGTTG	amplification of target *Dnl4*
P_Km01_018(deletion1(Ura3)_Fw)	CTAGCTCTAAAACCCTTTGATTAGTTTATGGGCGATTCGAACTGCGGACGTTG	amplification of target Ura3_Fw
P_Km01_019(deletion2(Ura3)_Rv)	CTAGCTCTAAAACAGAACAAGCAGAGGTTCGAGGATTCGAACTGCGGACGTTG	amplification of target Ura3_Rv
P_Km01_059(deletion2(Sed1)_Fw)	CTAGCTCTAAAACCTGGAATAGAACTGGATGTGGATTCGAACTGCGGACGTTG	amplification of target *Sed1*-2

Primer pairs for insert donor DNA

P_Km01_039(Ura3(5)_Fw)	ACCTTAAGTAGTCAAACAAATTGTG	amplification of GFP and bleomycin expression donor DNA containing length of 1000 bp *Ura3* homologous sequence (donor DNA-I)
P_Km01_046(Ura3(3)_Rv)	CAGAGAGTCTGCAACACCGA	
Ura3_up_100F	TGAATCTGAGCTCATCACCAAC	amplification of GFP and bleomycin expression donor DNA containing length of 122/109 bp *Ura3* homologous sequence (donor DNA-II)
Ura3_down100_R	GTATACAATGTGCGCAATGCC	
P_Km01_052(Ble_GFP_50bp_Rv)	TGGAACAGCAAGAGGAAGTATC	amplification of GFP expression donor DNA containing length of 50 bp *Ura3* homologous sequence (donor DNA-III)
P_Km01_053(GFP_50bp_Fw)	GATCAACTTGTAACTTCTGATCCGAGTACACTC GAACCTCTGCTTGTTCTgatcccttgtacagctcgtc	
P_Km01_060(GFP_Sed1_Fw)	TATTCGTGGTACCAGTTTTATTTGCATCATCTGC ATTTGCTGCTTTCTCTatggtgagcaagggcga	amplification of GFP codin region donor DNA containing length of 50 bp *Sed1* homologous sequence (donor DNA-IV)
P_Km01_061(GFP_Sed1_Rv)	gaaGATGGGGCAACTGAGGTCACtACTGGAATAG AACTGGATGTGTCGTTacccttgtacagctcgtcca	
P_Km01_052(Ble_GFP_50bp_Rv)	TGGAACAGCAAGAGGAAGTATC	amplification of P_{PDC1} region donor DNA containing length of 50 bp homologous sequence (donor DNA-V)
P_Km01_064(pPDC1_Fw)	atgggcaccaccccggtgaa	
P_Km01_065(EGFP_Rv)	GCTAGCatggtgagcaaggg	amplification of GFP coding region donor

P_Km01_066(EGFP_Fw)	AACTATACAAATGACAAGTTCTTGAAAACAAG AATCTTTTTATTGTCAGTgatcccttgtacagctcgtc	DNA containing length of 50 bp homologous sequence (donor DNA-VI)
P_Km01_051(Ble_GFP_50bp_Fw)	GATCAACTTGTAACTTCTGATCCG	amplification of T_{TEF1} region donor DNA containing length of 50 bp homologous sequence (donor DNA-VII)
P_Km01_067(TEF1t_Rv)	ACTGACAATAAAAAGATTCTTGT	

Red indicates each target sequence.
